# Assessing ambient air pollution’s effects on birth outcomes: a Scottish IVF cohort study (2010 -2018)

**DOI:** 10.1186/s12940-025-01204-4

**Published:** 2025-08-04

**Authors:** Haoze Song, Tom Clemens, Ruth M. Doherty, Jenny Stocker, Siladitya Bhattacharya

**Affiliations:** 1 University of Edinburgh, School of Geoscience, School of GeoSciences, University of Edinburgh, Edinburgh, UK; 2 Cambridge Environmental Research Consultants (United Kingdom), Cambridge, UK; 3 University of Aberdeen, Aberdeen, UK

**Keywords:** IVF, Air pollution, ADMS-Urban, Low birth weight, Preterm birth, SGA, IVF, ICSI

## Abstract

**Background:**

Ambient air pollution exposure during and before the pregnancy could result in adverse birth outcomes. This study uses data from women undergoing in vitro fertilization (IVF) data to investigate the associations between ambient air pollution exposure and adverse birth outcomes.

**Methods:**

This study analyses the associations between adverse birth outcomes, namely low birth weight (LBW), small for gestational age (SGA), and preterm birth and daily mean air pollution exposure during each of four IVF windows. The air pollutants considered were particulate matter with an aerodynamic diameter of less than 10 µm (PM_10_) and 2.5 µm (PM_2.5_), as well as nitrogen dioxide (NO_2_), which were estimated using the Atmospheric Dispersion Modelling System (ADMS-Urban). This data was linked to the IVF patients' postcode providing estimates of exposure to air pollutants. Logistic regression models were used to quantify the associations between air pollution exposure and adverse birth outcomes, and conditioning confounding factors. A subgroup analysis was conducted to investigate the differences in the effects of ambient air pollution exposure on the ICSI and IVF groups.

**Results:**

From January 2010 to May 2018, there are 2069 babies were able to be included in this study. We found no significant associations between air pollution exposure and the risk of adverse birth outcomes during window 1(85 days before oocyte retrieval) and 2 (14 days after gonadotrophin medication). With 1 µg⋅m^−3^ increase in PM_10_ concentration during window 3 (14 days after embryo transfer) and 4 (embryo transfer to delivery) led to a 5% (95% CI: 1.05—1.06) and 10% (95% CI: 1.01—1.21) increase in the odds of preterm birth, but not other outcomes. In window 3, every 1 µg⋅m^−3^ increase in NO_2_ concentrations resulted in a 2% (95% CI: 1.00 – 1.04) increase in the odds of LBW and a 3% (95% CI: 1.00 —1.05) increase in the odds of SGA but showed no effect for preterm birth. The results of the subgroup analysis suggest that the air pollution exposure may have a greater impact on the IVF group compared to the ICSI group.

**Conclusion:**

The results suggest that exposure to air pollution during the very early stage of pregnancy (14 days after conception) may represent the most critical window of susceptibility to an increased risk of adverse birth outcomes.

**Supplementary information:**

The online version contains supplementary material available at 10.1186/s12940-025-01204-4.

## Introduction

The global population undergoing assisted reproduction technology is expanding. According to the International Committee for Monitoring Assisted Reproductive Technologies (ICMART), the number of in vitro fertilization (IVF) cycles in Asia increased approximately threefold from 2017 (around 600,000 cycles) to 2019 (around 1800,000 cycles) [22]. However, studies on the impact of environmental factors on birth outcomes within this population are rare. Environmental exposures have been explored in naturally conceiving populations. For instance, Lee et al. [29] analysed 388,105 term births in South Korea, finding that exposure to PM_10_ and NO_2_ during the early stages of pregnancy was associated with low birth weight. Likewise, exposure to PM_2.5_ was associated with a 23% increase in the odds of small for gestational age (SGA) in a California population [43]. In Scotland, Clemens et al. [11] reported that PM_2.5_ exposure during the second trimester increased the risk of a reduction in biparietal diameter (BD), associated with limited fetal growth. Restricted fetal growth during the prenatal period is associated with an elevated risk of developing hypertension [4] and an increased mortality rate related to cardiovascular diseases in adult life [5, 12, 41, 63]. However, to the best of our knowledge, no study has investigated the effects of environmental factors on the risk of adverse birth outcomes **in** IVF populations.

Previous epidemiological studies have focused on the exposure during trimester-specific exposure windows in regards of the effects of air pollution on birth outcomes. However, there is a need to examine narrower exposure windows, as early gestational stages can also significantly influence fetal development and later health outcomes [13, 24, 46]. The placenta is a temporary organ that plays a critical role in transferring oxygen and essential nutrients between the mother and fetus. The development of the placenta is closely associated with fetal development. Black carbon (BC) has been detected in the human placenta tissue (Saenen et al., 2019). Furthermore, epidemiological studies have reported that exposure to air pollution during early pregnancy is associated with lower placental weights [49] and an increased risk of placental DNA methylation changes [17]. These changes are in turn linked to restricted fetal growth and an increased risk of adverse birth outcomes [25, 31].

In vitro fertilization (IVF) registries record precise dates for each IVF cycle, allowing for more accurate and narrow assessment of exposure during specific time frames including the exact date of conception.

Intracytoplasmic sperm injection (ICSI) was developed for patients with male-factor infertility [6]. In conventional IVF, collected eggs and sperm are mixed, allowing fertilization to occur naturally. In contrast, during ICSI, an embryologist selects a single sperm according to the sperm morphology and injects it into the egg to facilitate fertilization. However, due to suboptimal sperm quality and the bypassing of the natural sperm selection process, ICSI may be associated with a higher risk of malformations in children born through this technique [14]. Despite these concerns, the effects of environmental factors on birth outcomes following IVF/ICSI treatments remain understudied.

This paper aims to investigate the association between ambient air pollution exposure (PM_2.5_, PM_10_ and NO_2_) during the IVF process and subsequent adverse birth outcomes including SGA, preterm birth, and low birth weight from a 9-year cohort of the IVF population in Scotland. Additionally, we conducted a subgroup analysis to determine whether babies conceived via ICSI are more susceptible to air pollution exposure than those conceived through conventional IVF.

## Materials and methods

### Study area and the administrative data

**Fig. 1:**
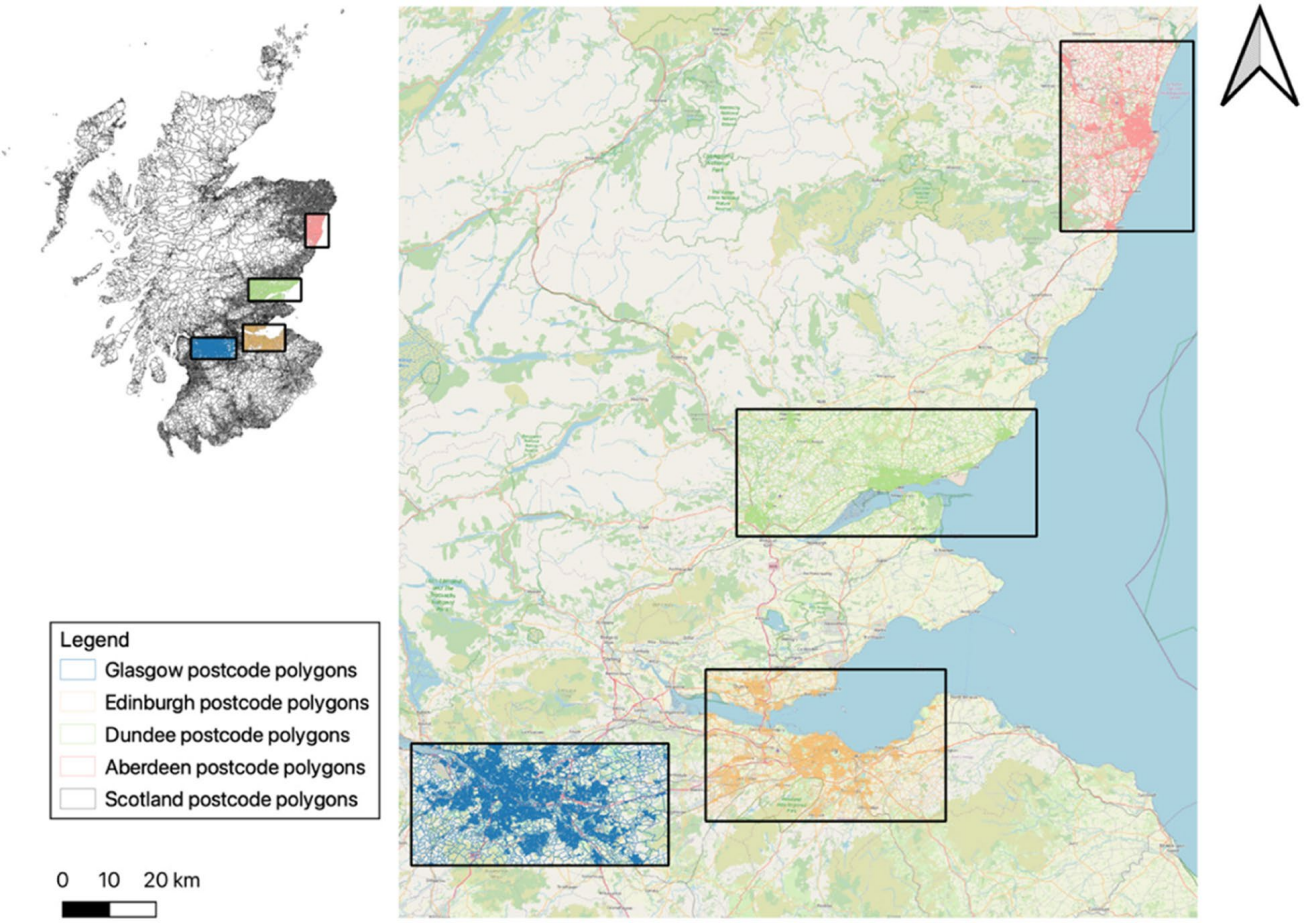
Studied regions in Scotland. For the postcode polygons, areas surrounding Glasgow, Edinburgh, Dundee, and Aberdeen are highlighted in blue, yellow, red, and green. Source: National Records of Scotland [37]. NRS Postcode polygon map. Background map © OpenStreetMap contributors [42]

The study population was drawn from several regions across Scotland (Fig. 1). The area surrounding Glasgow has a population of approximately 1,658,300, while the regions of Edinburgh, Dundee, and Aberdeen have populations of 1,030,170; 288,430; and 293,740, respectively [38]. These cities are the most densely populated in Scotland.

Due to data protection policies, researchers do not have access to individual patient addresses when performing statistical analyses. Therefore, selecting these urban centres enabled us to include the largest number of IVF patients in the exposure assessment. Additionally, these cities were chosen because they contain most Scotland’s air quality monitoring stations, which are essential for input and validation in the ADMS-Urban dispersion model set-up. Furthermore, air pollution concentrations exhibit significant spatial variability within urban areas, largely driven by human activities such as traffic emissions, industrial processes, and construction. This variability leads to differential exposure among residents, which in turn contributes to disparities in adverse health outcomes [30].

This study uses three databases: the UK Human Fertilisation and Embryology Authority (HFEA) IVF (including Intracytoplasmic sperm injection [ICSI] cases), Scottish morbidity records (SMR02), and the National Records of Scotland (NRS). Data from the HFEA, NRS, and SMR02 cover the period from January 2010 to May 2018. The HFEA, a regulatory body, maintains the UK national IVF database —commonly referred to as the HFEA register or the anonymized register. Under the Human Fertilisation and Embryology Act of 2008, every licensed fertility clinic in the UK is legally required to submit IVF treatment data to the HFEA. Typically, this data becomes available approximately two years after treatment completion, allowing time for clinics to report treatment outcomes and for data validation processes to be completed.

**Fig. 2:**
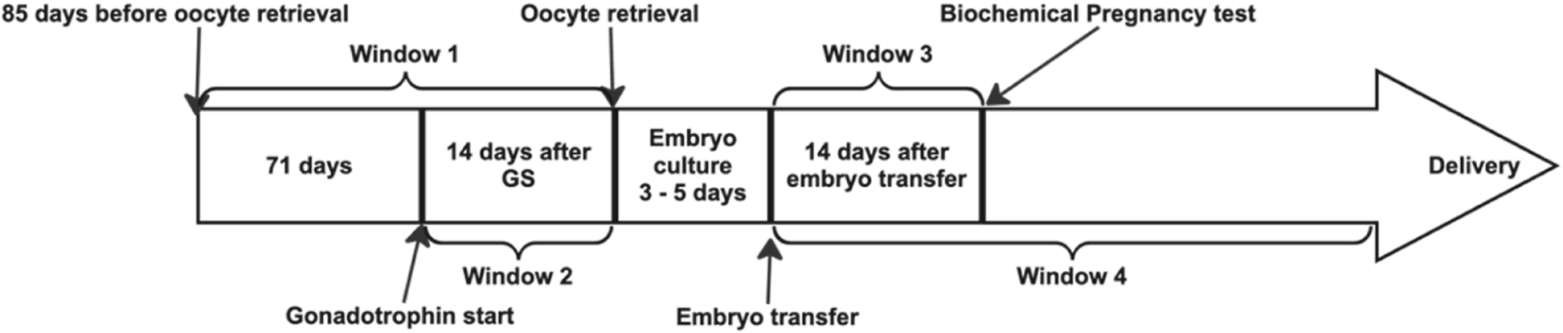
The exposure windows and process of IVF treatment. The studied windows are classified as follows: Window 1: 85 days before Oocyte retrieval. Window 2: 14 days from Gonadotrophin start to oocyte retrieval. Window 3: embryo transfer to biochemical pregnancy. Window 4: Embryo transfer to delivery

The IVF data recorded the date of gonadotrophin stimulation initiation, oocyte retrieval, embryo transfer, results of biochemical and clinical pregnancy test, and birth. The IVF stages and the designated exposure windows are depicted in Fig. 2. SMR02 and NRS data collects information on newborns and their parents, including smoking history and occupation social class. Each patient is assigned a unique identifier by their Scottish GP, known as the Community Health Index (CHI) number. This allows for the linkage of an individual’s health records with other databases in Scotland [1]. The study focuses on four time windows. Window 1 is 85 days before oocyte retrieval, representing one full cycle of follicle maturation [45]. Window 2 spans from the start of gonadotrophin simulation to oocyte retrieval. Greater air pollution exposure during window 1 and window 2 might be associated with a higher risk of poor oocyte quality [27] and could lead to an increased risk of birth defects [26]. Window 3 covers the 14 days after embryo transfer. In window 3, the placenta is in the very early stages of development [19]. Exposure during Window 3 may affect placental development, which could influence embryo development and contribute to restricted fetal growth [59]. Window 4 includes the gestation period, which averages 36 weeks in this cohort. These exposure windows have been used in previous research [15].

## Ethics approval

Ethical approval for this project was obtained from multiple bodies. Approval to the use fertility data was granted by the Human Fertilisation and Embryology Authority (HFEA) Register Research Panel (RRP) (HFEA, 2024). Access to Scottish health data and the Community Health Index (CHI) linkage service was reviewed and approved by the NHS Scotland Public Benefit and Privacy Panel for Health and Social Care (PBPP).

Following these approvals, all data access, linkage, and analysis were facilitated by the electronic Data Research and Innovation Service (eDRIS), maintained by Public Health Scotland (PHS). The project also received ethical approval from the University of Edinburgh’s Research Ethics Committee.

All data analyses were conducted within the National Safe Haven (NSH), a secure environment designed for handling sensitive data. The NSH is maintained by PHS. Researchers accessed the platform using University-managed devices, ensuring compliance with institutional and national data governance policies. Prior to publication, all results were reviewed by the designated eDRIS coordinator.

### Studied population

According to the National Infertility Group in Scotland [48], the IVF-born population accounts for approximately 3.5% of births annually in Scotland. From 2003 to 2023, Scotland recorded an average of 53,462 births per year [39], translating to roughly 1,603 IVF-conceived babies born annually.

The SMR02 data indicates that 9,018 IVF newborns were recorded in Scotland between January 2010 and May 2018. This figure is lower than expected, and the discrepancy can be attributed to changes in IVF legislation enacted in 2010, which altered how patients provide consent for the use of their data in research.

To protect patient confidentiality, the Human Fertilisation and Embryology (Disclosure of Information for Research Purposes) Regulations 2010 introduced an opt-out system. Patients who do not wish to share their data for research purposes can complete the ‘Consent for Disclosure’ (CD) form. When this policy was introduced by the HFEA in 2008, approximately 70% of patients opted out of data sharing. This percentage gradually declined to around 44% by 2018. Carson et al. [10] found that individuals in higher occupational social classes were more likely to consent to data sharing for research purposes.

### Data exclusion

The modelled air pollution data represent outdoor exposure levels linked to the nearest postcode for 2,000 IVF patients across the studied regions. Sixty IVF patients were excluded due to missing values, abnormal values (birthweights < 500 g), or stillbirths. For data confidentiality, specific statistics involving fewer than ten cases cannot be disclosed.

**Fig. 3:**
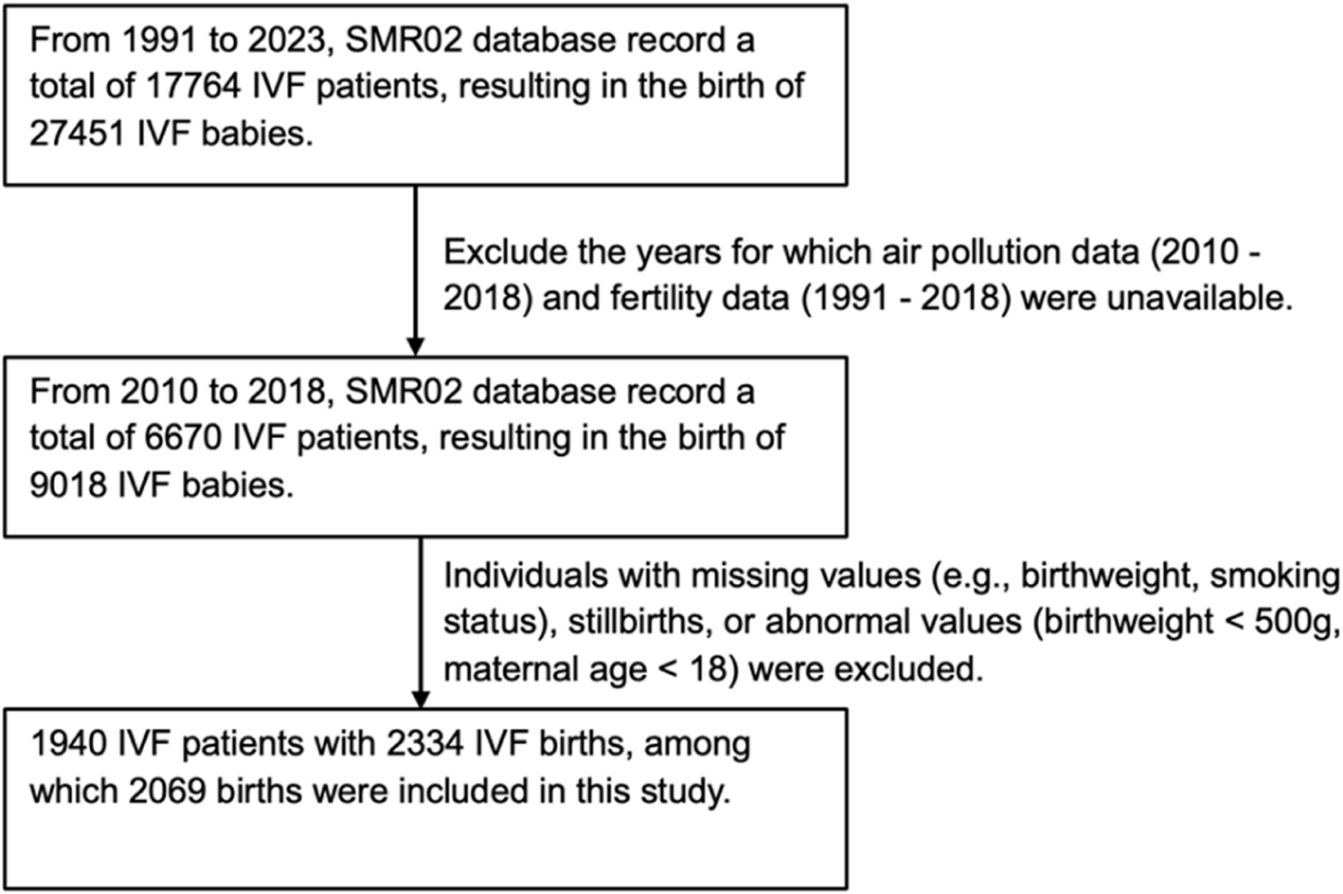
Flowchart of patients included in this study. SMR02: Scottish Morbidity Records. HFEA: UK Human Fertilisation and Embryology Authority

Only the first delivery per mother was included in the analysis. In cases of multiple births (e.g., twins or triplets) resulting from a single IVF cycle, these were not excluded; however, only data from the first-born infant of each delivery were used. If a mother had multiple deliveries from separate IVF cycles, each of those deliveries was eligible for inclusion. As a result, the final study population includes 2,069 IVF births from 1,940 IVF patients. The data exclusion process is summarised in Fig. 3.

### Outcome measures

Live infants with a birth weight under 2,500 g were categorised as low birth weight (LBW) [21]. Small for gestational age (SGA) was defined as a birth weight below the 10th percentile for the population’s birth weight distribution [35]. Preterm birth was identified when a live-born infant had a gestational age less than 37 weeks [8]. These outcomes were treated as categorical variables in our analyses.

### Air pollution data

Spatio-temporal daily mean air pollution concentrations were simulated using the Atmospheric Dispersion Modelling System (ADMS-Urban), developed by Cambridge Environmental Research Consultants (CERC). ADMS-Urban is based on Gaussian plume dispersion theory and incorporates a simplified chemical reaction scheme. The model uses gridded emissions data based on the National Atmospheric Emission Inventory (NAEI) (Appendix Table 1) to simulate street-scale daily PM_10_, PM_2.5_, and NO_2_ mass concentrations (unit = µg⋅m^−3^) from 2010 to 2019. The model accounts for local meteorological conditions, including wind speed and direction, temperature, relative humidity, precipitation, and cloud cover. Meteorological data were obtained from the nearest UK Met Office weather station, which is located at the local airport. The measurement height is 10 m above the ground. These data were accessed using the “worldmet” package in R, which is supported by the National Oceanic and Atmospheric Administration (NOAA).

The model simulates background air pollution levels to account for regional transport as well as air pollution from local emissions sources. Air pollutant concentrations are generated for specific averaging periods at pre-determined locations. In this study, these locations correspond to the centroids of postcode polygons, which represent patients’residential addresses, allowing for individualized exposure assessment. The use of modelled data enables linkage to postcode data rather than relying of measurements from ongoing fixed monitors for which there are typically only a few (< 20) per city in Scotland. To improve the accuracy of exposure assessment, receptor heights in the model are set at 1.6 m, approximating average human breathing levels.

The performance of the ADMS-Urban model was evaluated for each city by comparing modelled and observed air pollution concentrations. Summary statistics for the performance evaluation are presented in Table 1.

**Table 1:** Model performance statistic for each city (Units: µg·m⁻^3^). ABD, ED3, GLA5 and DUN1 is the selected monitoring stations, the details of these stations can be found: https://www.scottishairquality.scot/ [Accessed 1 st Jan 2025]

City	Year	Pollutant	Observed annual mean	Modelled annual mean	MB	R
Aberdeen (ABD)	2016	NO_2_	20.4	21.2	0.74	0.72
	2016	PM_10_	11.7	10.3	−1.4	0.41
	2016	PM_2.5_	5.7	5.4	−0.35	0.64
Edinburgh (ED3)	2016	NO_2_	19.9	19.7	−0.24	0.87
	2016	PM_10_	10.7	11.2	0.48	0.76
	2016	PM_2.5_	6.5	5.6	−0.92	0.82
Glasgow (GLA5)	2016	NO_2_	20.3	27.5	7.25	0.52
	2016	PM_10_	15.1	12.0	−3.13	0.68
	2019	PM_2.5_	6.5	6.7	0.2	0.94
Dundee (DUN1)	2018	NO_2_	12.3	11.7	−0.61	0.71
	2018	PM_10_	9.1	9.7	0.56	0.68
	2018	PM_2.5_	5.5	6.5	1	0.7

The mean bias (MB) measures how well the model predicts the magnitudes of observations. An MB greater than 0 indicates the model overpredicts air pollution concentrations, while an MB less than 0 means the model underestimates them. Pearson correlation coefficients (R) measure the model’s ability to capture variations in the observed data, with the ideal value being R = 1. Observed data refer to air pollution concentrations measured at selected monitoring sites, while modelled data are the concentrations predicted by the ADMS-Urban model at those same locations. The formulas used to calculate MB and R are provided in Eq. 1 and Eq. 2, where M represents modelled values and O represents observed values.

1 $$MB= \frac{1}{n}\sum\nolimits_{i=1}^{N}{M}_{i}-{O}_{i}$$

2 $$R= \frac{1}{(n-1)}\sum\nolimits_{i=1}^{n}\left(\frac{{M}_{i}- \overline{M}}{{\sigma }_{M}}\right)\left(\frac{{O}_{i}- \overline{O}}{{\sigma }_{O}}\right)$$

### Data linkage

**Appendix Fig. 1:**
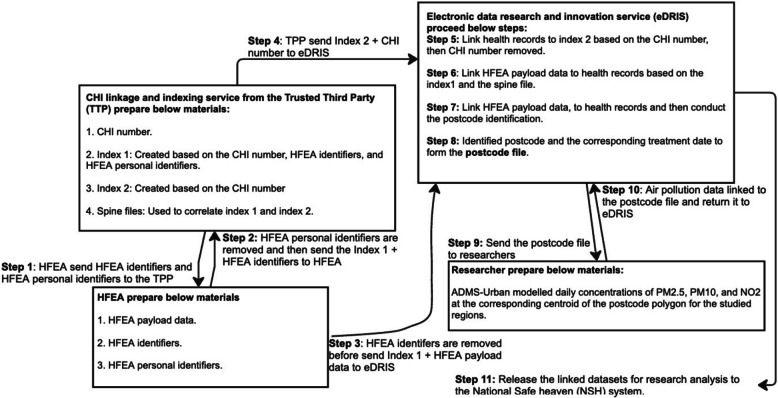
The process of linking the IVF data, Scotland health records (from SMR02 and national records), postcode and air pollution data

The specific linkage process is illustrated in Appendix Fig. 1. HFEA identifiers, including personal information such as date of birth, name, and age, along with the Community Health Index (CHI) number, are used to establish a connection between IVF data and Scottish health records.

Firstly, The HFEA submits these identifiers to the CHI Linkage and Indexing (CHILI) team, which is managed by the Public Health Scotland (PHS). In this context, the CHILI team acts as the Trusted Third Party (TTP). The CHILI team links the HFEA identifiers to the corresponding CHI number and generates a reference index—referred to as'Index 1'—which is then returned to the HFEA.

Following this, HFEA sends the payload data, accompanied by"index 1", to the electronic Data Research and Innovation Service (eDRIS). Meanwhile, the CHILI team creates a second reference index ("index 2") derived from the CHI number and assembles a'spine'file that maps index 1 to index 2. These compiled files are then securely transferred to eDRIS to facilitate the linkage of HFEA data with Scotland health data (SMR02 and NRS), ensuring that the CHI number and HFEA registry are not directly connected, thereby upholding the privacy of patient data. During this process, eDRIS carries out the linkage between pseudonymised HFEA data and pseudonymised Scottish health records. All linkages are conducted within the National Safe Haven (NSH), a secure platform managed by Public Health Scotland (PHS). The NSH provides a controlled environment where project-specific data are securely uploaded, stored, and accessed by approved researchers.

The linkage quality and matching procedures were overseen and validated by eDRIS coordinators, who followed standard protocols to ensure high-quality matching. However, due to data protection policies, exact measures of linkage error (e.g., unmatched records) could not be disclosed.

Subsequently, the eDRIS coordinator identifies the patients'residential postcodes. These postcodes, along with corresponding treatment dates, are then provided to researchers. Utilising these two data points, researchers with appropriate permissions are able to correlate air pollution concentrations with the specific postcodes and treatment dates. Researchers perform this process via university managed devices. Individuals'exposure is calculated based on their varied treatment dates. The linked file is then returned to eDRIS to remove the postcode before processing the linkage between the air pollution exposure data, fertility data, and Scottish health records. Lastly, researchers can access and analyse the linked data through the National Safe Haven.

### Statistical analysis and covariates selection

To investigate the associations between adverse birth outcomes following IVF and ambient air pollution exposure, we employed multivariable logistic regression models. Both crude and confounder-adjusted analyses were performed. The crude analysis used only air pollution concentrations as predictors, while the adjusted models included confounding factors identified by Directed Acyclic Graphs (DAGs).

**Fig. 4:**
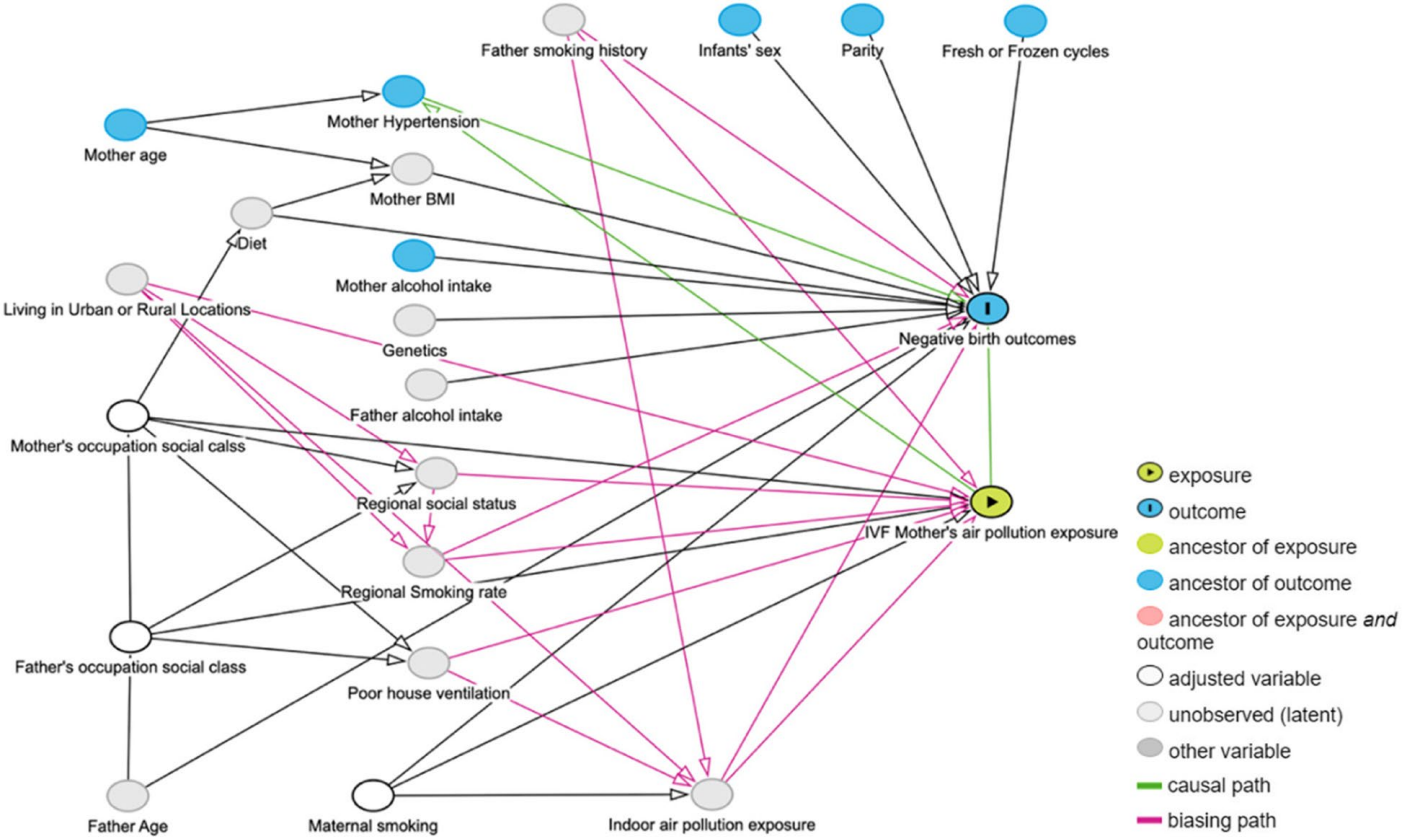
Directed acyclic graph for the relationship between air pollution exposure and adverse birth outcomes. This figure illustrating key confounding factors that identified by researchers in the association between air pollution and adverse birth outcomes. The identified confounding variables are the nodes connected to the causal paths (highlighted in pink colour). The confounding factors include the patient’s address (urban or rural), the occupation social class of both parents, the smoking history of both mother and father, and exposure to indoor air pollution. The confounding factors were selected based on the DAG and the availability of the variables. The adjusted variables are mother’s occupation social class and Mother smoking during pregnancy

DAGs were used to graphically represent and assess the potential causal relationships between air pollution, birth outcomes, and other covariates. The theoretical framework for this approach is detailed by Tennant et al. [53]. The most common source of confounding is from factors that could influence both air pollution and birth outcomes. Other more complex sources of confounding can also be present which a DAG can help to identify confounding factors. We constructed a DAG (Fig. 4) using the Dagitty platform [52] and a priori knowledge to assess how lifestyle factors might influence both air pollution exposure levels and birth outcomes in this context. Based on this analysis, we identified the following confounding factors: living locations (urban or rural), maternity smoking (yes or no), mothers’ occupation social class (0 ~ 9), fathers’ smoking status, and indoor generated air pollution concentrations. The available covariates are maternity smoking (yes or no), mothers’ occupational social class (0 ~ 9), and those variables were retained to adjust the multivariable logistic regression model. Mother’s social class is categorised in the National Records of Scotland (NRS) database across ten levels, ranging from 0 to 9. The Code description is shown in the Appendix Table 2.

To ensure the robustness of the findings, a separate model was developed that included conventional covariates known to influence birth outcomes, in addition to those identified via the DAG-based confounder selection. For the sensitivity analysis, the additional covariates, including parity [16], infants’ sex [55], and types of treatment (fresh or frozen) [32], were used to adjust the additional multivariable logistic regression model.

### Cluster standard errors adjustment for patients with multiple IVF/ICSI births

Some patients had more than one successful IVF birth, leading to the non-independence of observations within clusters, and resulting in biased standard errors [34]. The clustering issue arises due to repeated measurements over time within the same patients [18]. Clustered standard errors are used to address this problem, and the standard error adjustment is performed in R using the “sandwich” package.

### Subgroup analysis

To compare the effects of air pollution exposure on different IVF treatments, the population was divided into two groups: conventional IVF and ICSI. Both crude and confounder-adjusted analyses were performed on these groups.

## Results

### Population characteristics

This cohort included 1,940 patients. Among them, 127 patients have had more than two successful IVF treatments. A total of 2,069 IVF births were included in this study. The characteristics of the mothers and their birth outcomes included in this study are summarised in Tables 2 and 3. A total of 804 patients underwent conventional IVF, while 857 patients received ICSI treatment. There were 835 IVF-conceived babies and 888 ICSI-conceived babies, respectively. The treatment type (IVF or ICSI) for 279 patients was not recorded. The average maternal age was 35 years for both the overall group and the conventional IVF group. The average age of the ICSI group was 34 years.

**Table 2:** Demographic characteristics of studied patients (with confidentiality considerations). The parents’ occupation social class code explanation is shown in the Appendix Table 2. The exact proportions for each social class level cannot be disclosed due to data confidentiality rules

Characteristics of patients	Total	IVF	ICSI
Number of mothers underwent IVF/ICSI	1,940	804	857
Number of births	2069	835	888
Age (mean ± SD)	35 ± 4	35 ± 4	34 ± 4
Total attempts	2854	1327	1527
Cycle types			
Number of fresh cycles	1537 (74%)	719 (86%)	814 (92%)
Number of frozen cycles	532 (26%)	116 (14%)	74 (8.3%)
Unknown	341		
Gestational weeks (mean ± SD)	36 ± 3	36 ± 2	36 ± 3
Parity			
0	1495	558	717
> = 1	559	268	167
Number of embryos transferred (n (%))			
1	937 (48%)	406(48%)	410(46%)
2	968 (50%)	405(48%)	464(52%)
> = 3	35 (2%)	24(2.9%)	11(1.6%)
Diabetes (n (%))			
Yes	1855 (96%)	767 (96%)	821 (98%)
NO	55 (3%)	27 (3.4%)	19 (2.3%)
Hypertension (n (%))	83 (4.3%)	37 (4.6%)	28 (3.2%)
Smoking history (n (%))			
Never smoked	1649 (85%)	650 (82%)	720 (86%)
Current smoker	40 (2%)	22 (2.8%)	18 (2.2%)
Former smoker	210(13%)	116 (15%)	96 (12%)
Maternal smoking (n (%))			
No	1837 (97%)	764 (97%)	811 (98%)
Yes	48 (2.5%)	21 (2.7%)	16 (2%)
Father’s social class (n (%))			
0–3	1272 (67.3%)	584 (71%)	569(64.2%)
4–6	464 (24.3%)	183(22%)	214(24.2%)
7—9	174 (9.1%)	66(7.9%)	101(11.4%)
Mother’s social class (n (%))			
0–3	1499 (81.7%)	658 (78.1%)	518 (56%)
4–6	269 (14.8%)	110 (13.2%)	286 (13.1%)
7—9	58 (3.2%)	65 (7.7%)	91 (5.9%)

**Table 3:** Characteristics of IVF and ICSI births

Characteristics of births	Total	IVF	ICSI
Birth weight (mean ± SD)	3,211 ± 685	3,223 ± 678	3,176 ± 667
Male infertility (n (%))			
No	601 (31%)	442 (53%)	195 (22%)
Yes	1280 (66%)	275 (33%)	684 (77%)
Low birth weight (n (%))			
No	1678(87%)	723(87%)	773(87%)
Yes	255(13%)	112(13%)	115(13%)
Preterm birth (n (%))			
No	1045(54%)	479(58%)	456(52%)
Yes	878(46%)	352(42%)	429(48%)
SGA (n (%))			
NO	1925 (93%)	784(94%)	827(93%)
Yes	145 (7%)	51(6.1%)	61(6.9%)

Only 2.5% of the patients smoked during their pregnancies and 85% had never smoked. Approximately 81.7% of the mothers and 67.3% of the fathers were employed in professional occupations which corresponded to the findings related to social class from Carson et al., [10]. The IVF and ICSI groups demonstrated similar characteristics in terms of smoking history, age, birthweights, and the proportion of low birthweight, preterm birth and SGA. However, in the IVF group, 78% of patients belonged to social classes 0–3, with a higher proportion of patients in higher occupational classes compared to the ICSI group, where 56% of patients were in social classes 0–3.

Some patients underwent frozen cycles, resulting in varied exposure windows; for example, patients who underwent frozen IVF cycles did not have exposure during IVF window 1 (85 days before oocyte retrieval). Associations between ambient air pollution exposure and IVF birth outcomes, including low birth weight (LBW), SGA, and preterm birth, were analysed using both crude and confounder adjusted logistic regression models. As stated in statistic and covariables selection section, the mothers’ occupation social class, maternal smoking, were treated as confounding factors in the adjusted logistic regression model.

### Exposure characteristics

Table 4 presents the characteristics of assigned PM_10_, PM_2.5_, and NO_2_ concentrations for the IVF patients by different IVF windows. NO_2_ concentrations demonstrate substantial variability among IVF patients than that of particulate matters, for instance, in window 2 (14 days) and 4 (averagely 252 days), the standard deviation of NO_2_ concentrations reaches 6.1 µg⋅m^−3^ and 7.6 µg⋅m^−3^ respectively. PM_2.5_ and PM_10_ concentrations exhibit less variability, with the standard deviations of 1.4 µg⋅m^−3^ to 1.5 µg⋅m^−3^, respectively.

**Table 4:** Summary of estimated air pollution exposure for patients by windows (µg·m^−3^). Exposure is estimated by the daily mean air pollution concentrations using the ADMS-Urban model, and individuals'exposure is calculated based on their varied treatment dates

**Window**	PM_2.5_ (µg⋅m^−3^) Mean ± SD (min, max)	PM_10_(µg⋅m^−3^) Mean ± SD (min, max)	NO_2_(µg⋅m^−3^) Mean ± SD (min, max)
Window 1	6.3 ± 1.4 (3.4, 12.6)	9.5 ± 1.3 (6.5, 18.0)	15.7 ± 6.2 (4.5, 76.3)
Window 2	6.4 ± 2.2 (3.0, 17.4)	9.6 ± 2.4 (4.8, 21.5)	16.1 ± 7.6 (3.2, 76.3)
Window 3	6.3 ± 2.2 (3.0, 16.8)	9.5 ± 2.4 (4.7, 21.7)	15.5 ± 7.3 (2.5, 62.9)
Window 4	6.2 ± 1.4 (3.3, 13.7)	9.4 ± 1.5 (4.8, 24.5)	15.2 ± 6.1 (4.7, 90.6)

### Overall population analysis for the associations between air pollution exposure and birth outcomes

**Fig. 5:**
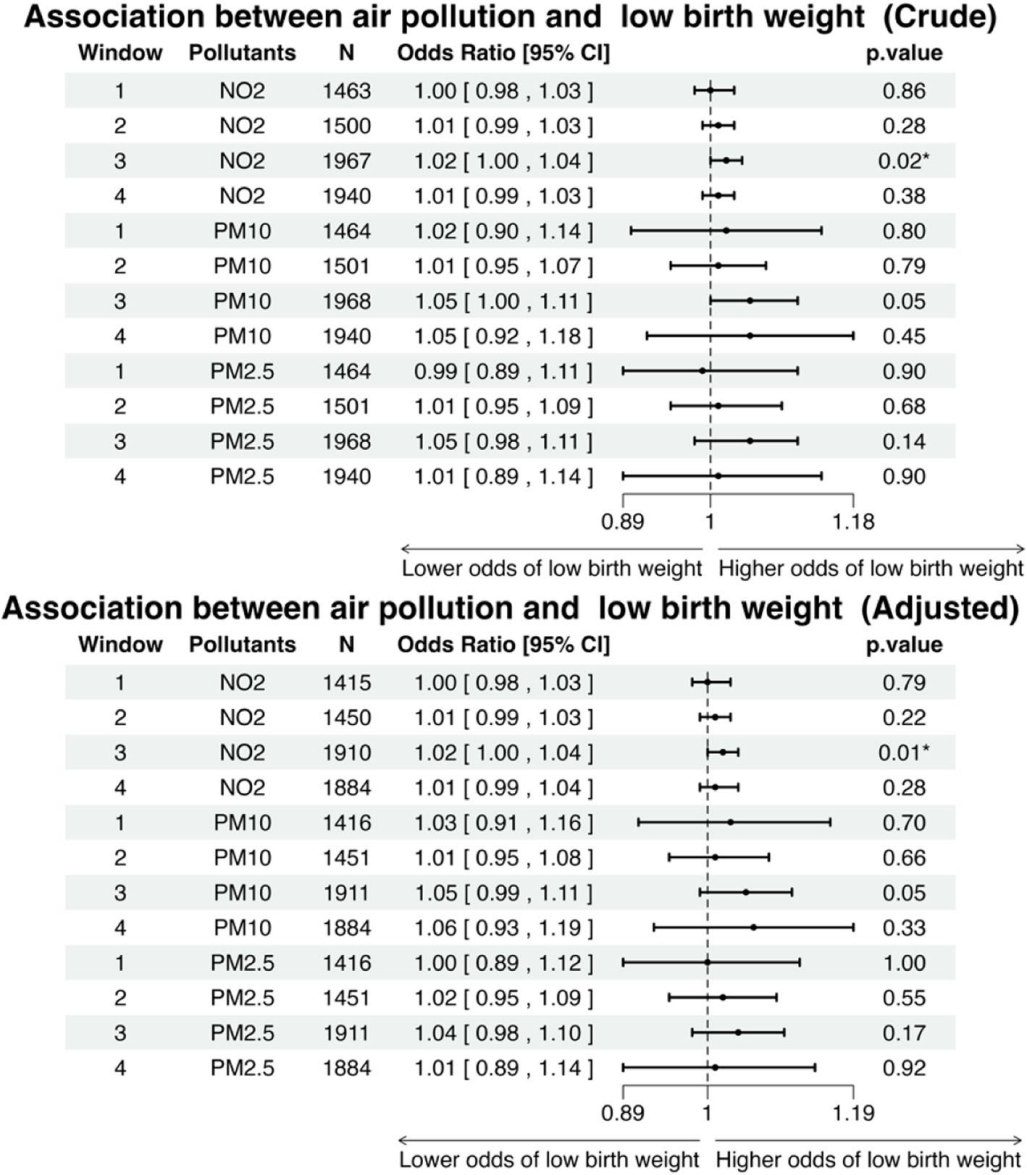
Forest plot showing the crude (top) and adjusted (bottom) odds ratios (ORs) with 95% confidence intervals (CIs) and *p*-values for the association between each 1 µg·m.^−3^ increase in air pollution concentrations and the odds of low birth weight across different exposure windows."‘*’ and ‘**’ indicate *p*-values less than 0.05 and 0.01, respectively. N refers to the number of cases included in the analysis. The adjusted variables include the mother's occupational social class (0—9) and smoking status during pregnancy (1 or 0)

Figure 5 presents the associations between air pollution exposure and the risk of LBW for the overall population. The crude and adjusted analysis for window 1(85 days before oocyte retrieval), window 2 (14 days after gonadotrophin medication) and window 4 (embryo transfer to delivery) showed that none of the air pollutants had statistically significant associations with the risk of LBW. During window 3, the crude and adjusted results indicated that every 1 µg⋅m^−3^ increase in NO_2_ concentrations resulted in a 2% increase in the odds of LBW. The effect size remained similar after adjusted analysis. Although the PM_10_ and PM_2.5_ were not statistically associated with the risk of LBW in window 3, the odds ratio suggests a potential positive relationship with risk of LBW. Adjusted model with additional covariates showed similar results (Supplementary Fig. 1).

**Fig. 6:**
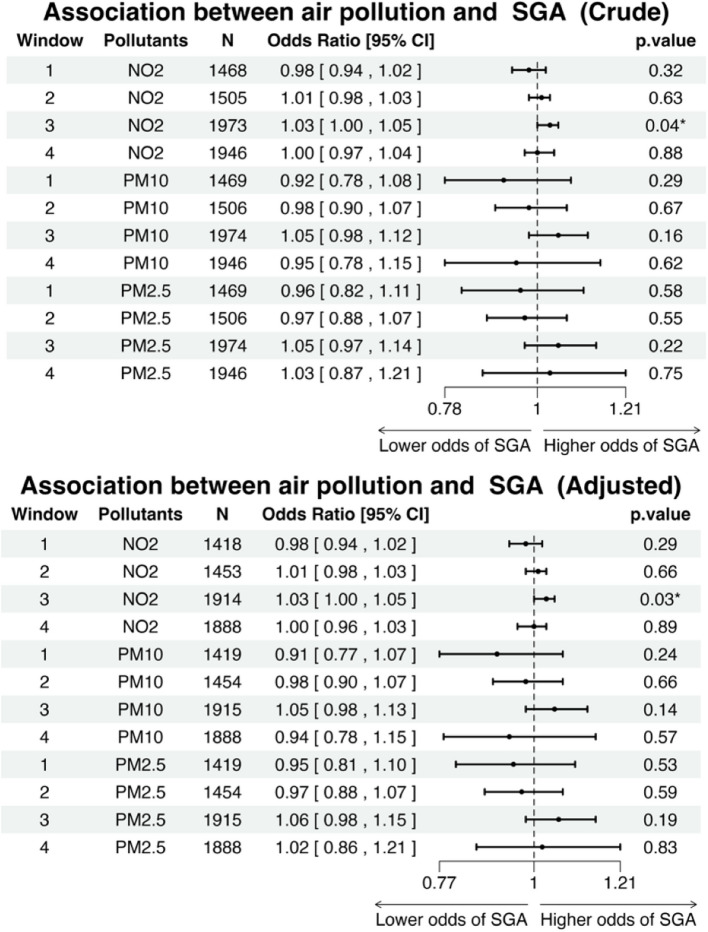
Forest plot showing the crude and adjusted odds ratios (ORs) with 95% confidence intervals (CIs) and *p*-values for the association between each 1 µg·m.^−3^ increase in air pollution concentrations and the odds of small in gestational age (SGA) across different exposure windows."‘*’ and ‘**’ indicate *p*-values less than 0.05 and 0.01, respectively. N refers to the number of cases included in the analysis. The adjusted variables include the mother's occupational social class (0—9) and smoking status during pregnancy (1 or 0)

**Appendix Fig. 2:**
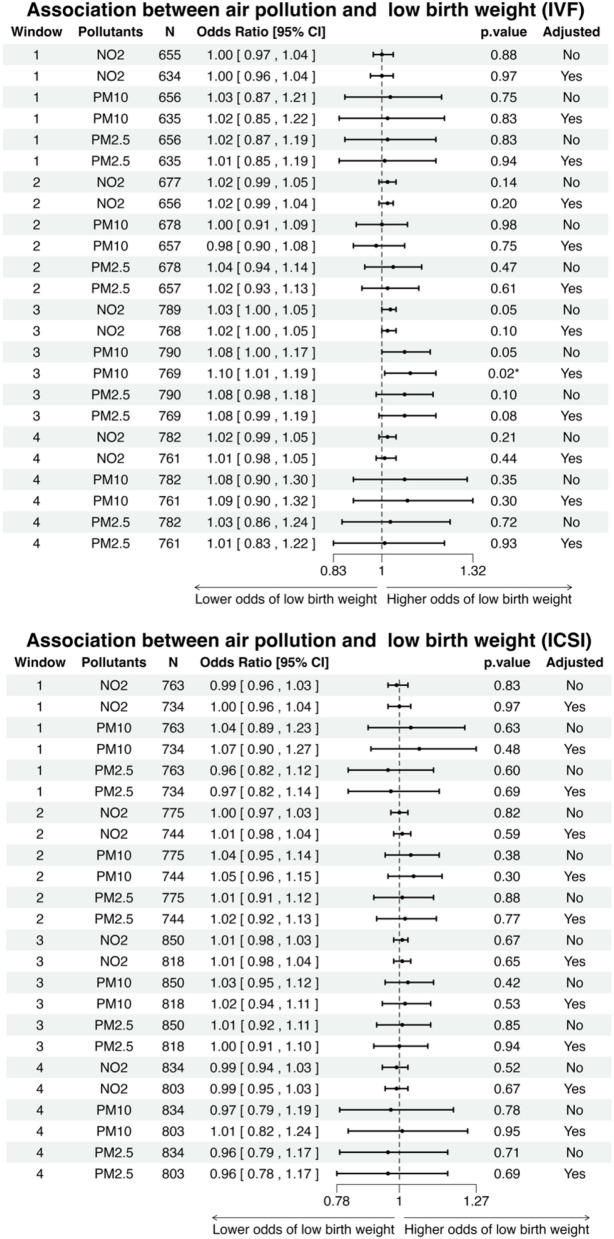
Subgroup analysis forest plot showing the crude and adjusted odds ratios (ORs) with 95% confidence intervals (CIs) and p-values for the association between each 1µg×m^-3^increase in air pollution concentrations and the odds of low birth weight across different exposure windows."‘*’ and ‘**’ indicate p-values less than 0.05 and 0.01, respectively. N refers to the number of cases included in the analysis. The adjusted variables include the mother's occupational social class (0 - 9) and smoking status during pregnancy (1 or 0)

Similarly, all air pollutants demonstrated a consistent association with the risk of Small for Gestational Age (SGA) (Fig. 6). In windows 1, 2, and 4, both crude and adjusted analysis, air pollutants were not statistically associated with the odds of SGA. However, both crude and adjusted model results indicated that NO_2_ exhibited a statistically significant positive association with higher odds of SGA, increasing the odds by 3% for each unit increase in NO_2_ concentrations. For SGA, the adjusted model with additional covariates showed similar results (Supplementary Fig. 2).

**Fig. 7:**
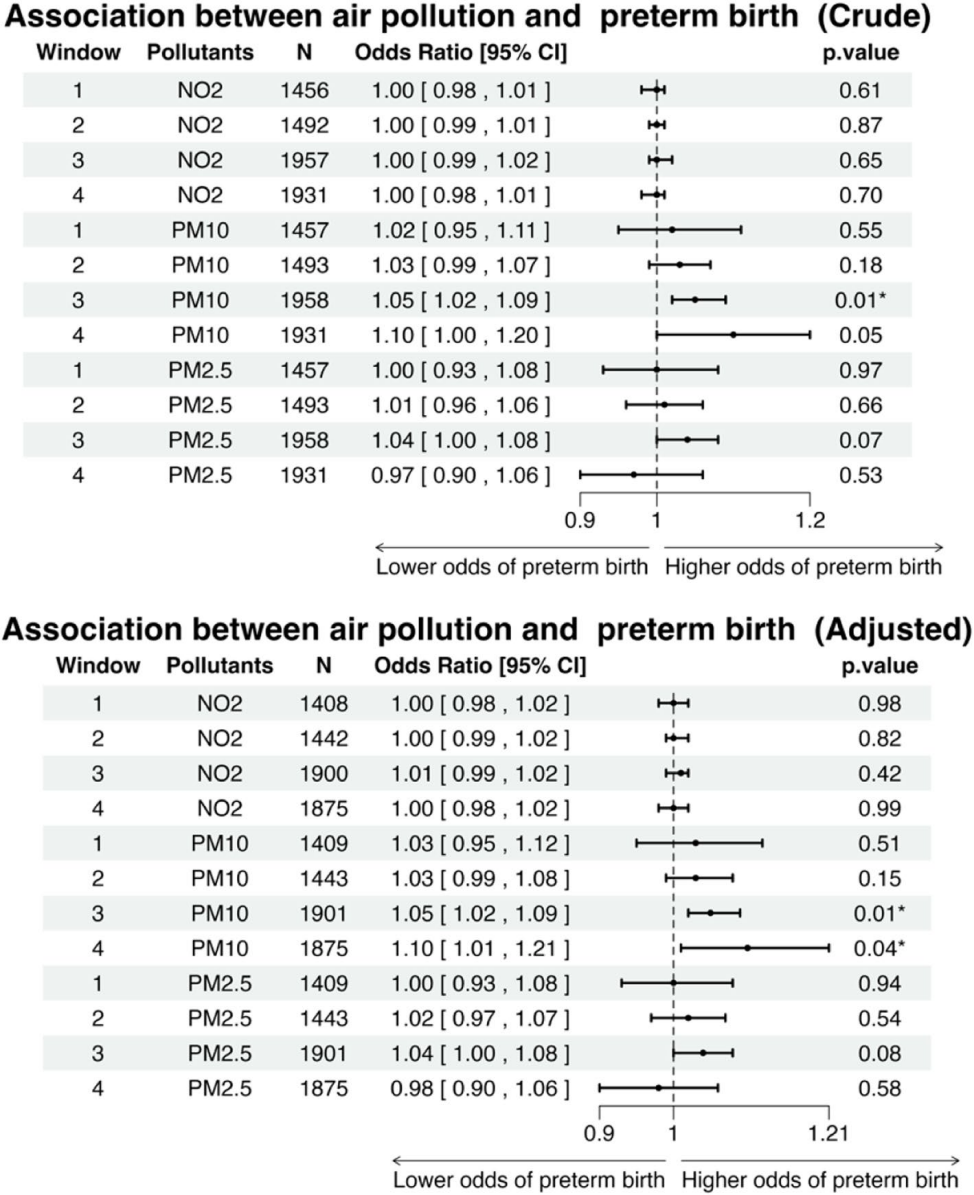
Forest plot showing the crude and adjusted odds ratios (ORs) with 95% confidence intervals (CIs) and *p*-values for the association between each 1 µg⋅m.^−3^ increase in air pollution concentrations and the odds of preterm birth across different exposure windows."‘*’ and ‘**’ indicate *p*-values less than 0.05 and 0.01, respectively. N refers to the number of cases included in the *analysis. The adjusted variables include the mother's occupational social class (0—9) and smoking status during pregnancy (1 or 0)*

**Appendix Fig. 3:**
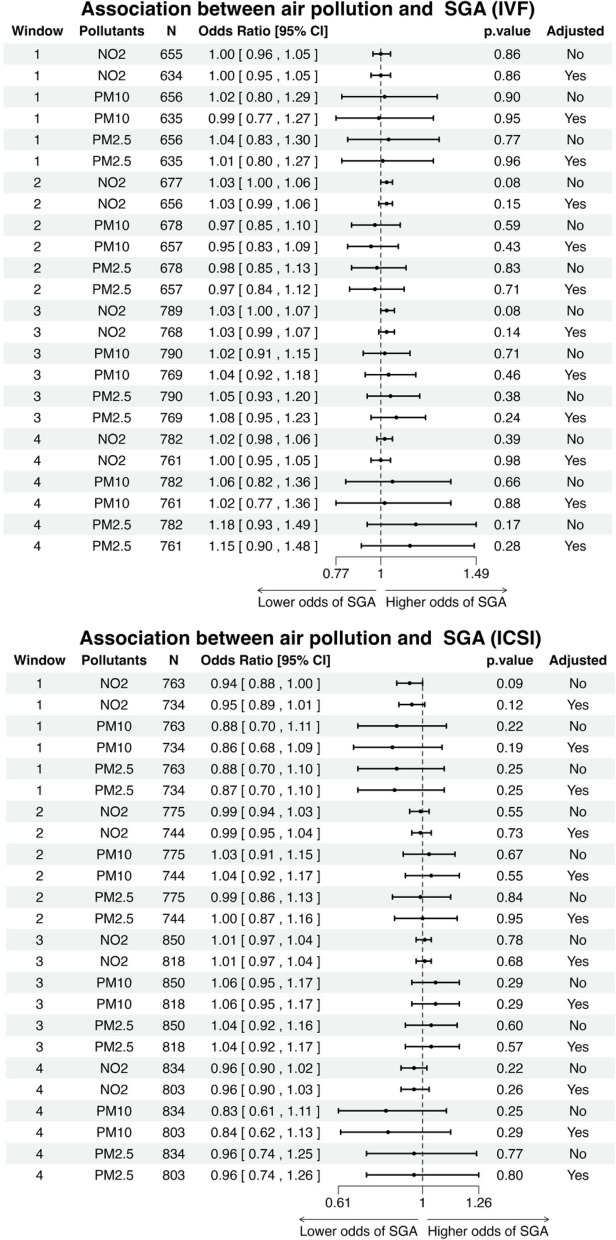
Subgroup analysis forest plot showing the crude and adjusted odds ratios (ORs) with 95% confidence intervals (CIs) and p-values for the association between each 1µg×m^-3^increase in air pollution concentrations and the odds of preterm birth across different exposure windows."‘*’ and ‘**’ indicate p-values less than 0.05 and 0.01, respectively. N refers to the number of cases included in the analysis. The adjusted variables include the mother's occupational social class (0 - 9) and smoking status during pregnancy (1 or 0)

For preterm birth (Fig. 7), air pollution exposure during windows 1 and 2 showed no significant association with the risk of preterm birth. PM_10_ exposure during window 3 was positively associated with the odds of preterm birth. Both crude (crude odds ratio = 1.05, 95% CI: 1.01–1.09) and adjusted (adjusted odds ratio = 1.05, 95% CI: 1.01–1.09) analyses showed that higher PM_10_ exposure led to a higher risk of preterm birth. There were no significant effects resulting from PM_2.5_ and NO_2_ exposure across all windows. In window 4, after the model adjustment, PM_10_ showed significant effects to the risk of preterm birth with 1 µg⋅m^−3^ increase in the PM_10_ concentrations, 10% (95% CI: 1.01–1.21) increase in odds of preterm birth. The adjusted model, which included covariates such as parity, infants'sex, and the types of IVF/ICSI cycles, provided results similar to those of the Directed Acyclic Graph (DAG) model (Supplementary Fig. 3).

### Subgroup analysis results

For low birth weight (Appendix Fig. 2), similar to the results observed in the overall population, in windows 1, 2, and 4, none of the pollutants showed significant effects on the odds of low birth weight in windows 1, 2, and 4. In window 3, the effects of air pollution on the IVF group were similar to those in the general population. Both crude and confounder-adjusted results indicated a positive relationship between air pollution concentrations and the odds of low birth weight. In window 3, for IVF group, every 1 µg⋅m^−3^ increase in PM_10_ was associated with a 10% increase in the odds of low birth weight (adjusted odds ratio = 1.10, 95% CI: 1.01—1.19). Both crude and adjusted analyses indicated that concentrations of PM_2.5_ and NO_2_ were also positively associated with the odds of low birth weight in window 3. However, for the ICSI group, all pollutants showed no statistically significant effects on the odds of low birth weight across all windows.

For SGA (Appendix Fig. 3), in window 1, none of the pollutants had significant effects on SGA in the IVF group, while they were associated with lower odds of SGA in the ICSI group. In window 2, NO_2_ concentrations increased the odds of SGA in the IVF group but had no significant effects in the ICSI group. PM_10_ and PM_2.5_ were linked to lower odds of SGA in the IVF group but higher odds in the ICSI group. In window 3, all pollutants were positively associated with higher odds of SGA in both subgroups, similar to the findings in overall population. In window 4, both crude and adjusted results indicated that PM_10_ and PM_2.5_ were positively associated with SGA in the IVF group but negatively associated in the ICSI group.

**Appendix Fig. 4:**
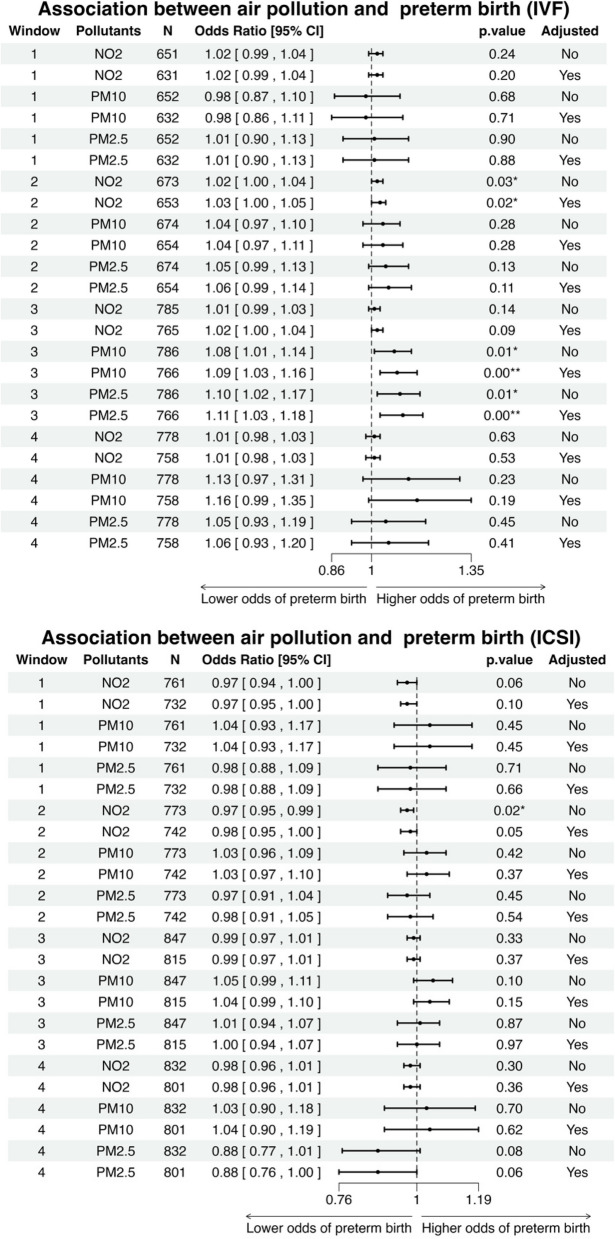
Subgroup analysis forest plot showing the crude and adjusted odds ratios (ORs) with 95% confidence intervals (CIs) and p-values for the association between each 1µg×m^-3^increase in air pollution concentrations and the odds of preterm birth across different exposure windows."‘*’ and ‘**’ indicate p-values less than 0.05 and 0.01, respectively. N refers to the number of cases included in the analysis. The adjusted variables include the mother's occupational social class (0 - 9) and smoking status during pregnancy (1 or 0)

For preterm birth (Appendix Fig. 4), NO_2_ did not have a significant effect on the odds of preterm birth in the overall population analysis. However, in subgroup analysis, NO_2_ was associated with higher odds of preterm birth in the IVF group but lower odds of preterm birth in the ICSI group in all windows. In window 1, PM_10_ and PM_2.5_ had no significant effect on odds of preterm birth, consistent with the overall population. In window 2, all pollutants were positively associated with odds of preterm birth in the IVF group. Adjusted and crude analyses showed that each 1 µg⋅m^−3^ increase in NO_2_ was associated with a 3% increase (adjusted odds ratio = 1.03, 1.00–1.05) and a 2% (crude odds ratio = 1.02, 1.00–1.04) increase in the odds of preterm birth in the IVF group, while NO_2_ was negatively associated with preterm birth in the ICSI group (adjusted odds ratio = 0.98, 95% CI: 0.95–1.00).

In window 3, PM_10_ (adjusted odds ratio = 1.09, 1.03–1.16) and PM_2.5_ (adjusted odds ratio = 1.11, 1.03–1.18) were strongly associated with higher odds of preterm birth in the IVF group, while both pollutants also positively correlated with preterm birth in the ICSI group. In window 4, PM_10_ and PM_2.5_ were positively related with preterm birth odds, but in the ICSI group, PM_10_ had no significant effect, and PM_2.5_ was negatively associated with odds of preterm birth.

## Discussion

### Main findings

The results of this study indicate that low birth weight (LBW), and Small for Gestational Age (SGA) were associated with exposure to higher levels of NO_2_ during the 14 days following embryo transfer (window 3). PM_10_ exposure during window 3 and 4 (embryo transfer to birth) was significantly associated with higher odds of preterm birth. PM_2.5_ exposure during window 3 might have adverse impacts on birth outcomes. No significant associations were found between air pollution exposure and the risk of adverse birth outcomes during windows 1 and 2.

### Study strengths and weaknesses

Our study has several strengths. Regarding exposure assessment, advances in individual-level exposure assessment using ADMS-Urban model have improved the accuracy of exposure data by providing estimates at the postcode level, rather than relying on a limited number of fixed monitors within a city region. In terms of confounding adjustment, we employed Directed Acyclic Graphs (DAGs) to illustrate the process. This method facilitates the visual mapping of relationships and potential confounding paths, thereby guiding an analytical strategy in a transparent manner [57]. Consequently, it prevents the addition of excessive covariates merely to achieve statistical significance. Additionally, the use of an IVF dataset enabled a more precise examination of the effects of air pollution within narrower exposure windows, potentially leading to more accurate correlations with birth outcomes.

However, there are several limitations to this study. For the exposure assessment, our analysis did not account for participants’ mobility or indoor air pollution exposure, as it relied solely on ambient pollution levels at their residential postcode. Due to the absence of individual-level data on time-activity patterns or workplace locations, the modelled outdoor NO₂ concentrations at the residential address may not accurately represent true personal exposure. This limitation may lead to exposure misclassification, potentially biasing the estimated effects. Empirical and simulation studies [7, 50] have shown that such misclassification typically attenuates the magnitude of effect estimates.

Multiple births were not excluded from the analysis, which may introduce bias, as twins and triplets are at a higher risk of low birthweight [33, 58]. The studied samples might include more of patients with higher level of occupational social class. These patients might have better medical resources and healthier lifestyles, which are not representative to the entire population. This introduces selection bias that may underestimate the true health impact of exposure, potentially reducing the external validity of our findings, particularly for lower socioeconomic groups who may experience greater vulnerability [54]. Further research with larger datasets from different population groups is needed.

We may have missed some important covariates due to the lack of variables, which could cause a DAG to fail to identify all confounding factors. Furthermore, the analysis of associations between outcomes and air pollution exposure was conducted four times using the same subjects across four different windows. This repetitive testing, including the subgroup analysis, introduces multiple testing concerns, and this can inherently inflate the risk of Type I errors, where false positives may arise from repeated hypothesis testing [28]. Lastly, the small number of cases in our study are not sufficient to draw definitive conclusions. However, findings for NO_2_ demonstrated consistent statistical significance in window 3, suggesting it might be a critical exposure period. Similarly, PM_10_ and PM_2.5_ exposure during this window was also linked to a higher risk of adverse birth outcomes, further indicating the potential vulnerability of this window to air pollution exposure.

### Relevance of findings to the literature

The PM_2.5_ exposure positively associated with the crude and adjusted odds of low birth weight (in window 2,3 and 4), preterm birth (in window 2 and 3) and SGA (in window 3 and 4), although the results are not statistically significant. In this case, a possible reason is that the air pollution levels in Scotland are relatively low, and the patients’ average PM_2.5_ exposure levels between those windows range from 6.2 to 6.4 µg⋅m^−3^. Tapia et al. [51] investigated the associations between PM_2.5_ exposure and adverse birth outcomes in Lima, Peru. The results indicated that with a 9.24 µg·m⁻^3^ increase in PM_2.5_ exposure during the full gestation period, the odds of LBW increased by 11%. Additionally, Ho et al. [20] reported that during the first month of pregnancy, exposure to higher levels of PM_2.5_ was associated with a 3% higher risk of preterm birth in the Vietnam population.

For PM_10_, our study found that exposure during Window 3 (14 days after embryo transfer) and Window 4 (embryo transfer to delivery) significantly increased the risk of preterm birth, with no significant effects on the risk of low birth weight (LBW) and small for gestational age (SGA) during any exposure windows. Similarly, van den Hooven et al. [56] found significant associations between PM_10_ and NO_2_ exposure during gestational periods and preterm birth in a cohort of 7,772 subjects in the Netherlands, with PM_10_ showing no significant effects on the risk of SGA. However, a large-scale study of 100,595 singletons found that PM_10_ exposure during the first trimester significantly increased the risk of term LBW [60].

In our study, NO_2_ was positively affected the risk of LBW and preterm birth during window 3 (full gestation). Likewise, Brauer et al. (2003) investigated the effects of air pollution exposure throughout pregnancy on birth outcomes. Their findings suggested that every 10 µg⋅m^−3^ increase in NO_2_ concentrations, as modelled by the land use regression (LUR) approach at an individual’s postcode region, decreased the risk of term LBW by 3%. Melody et al. [35] reported that long-term exposure to NO_2_ increased the risk of SGA by 6% (95% CI: 1.01–1.10).

Embryologists are concerned that the ICSI treatment may damage the oocyte membrane and introduce sperm with DNA defects, potentially leading to a higher risk of congenital abnormalities [62]. Studies have reported inconsistent findings regarding birth defects between the IVF and ICSI groups. Bonduelle et al. [9], using follow-up data, found that the risk of adverse birth outcomes (low birth weight, preterm birth, and malformations) was similar between 2,995 infants born after IVF and 2,889 infants born after ICSI. Furthermore, sperm quality was not associated with the risk of congenital malformations in children conceived through ICSI. In contrast, Xiong et al. [61], using administrative data, reported that the ICSI group exhibited a higher risk of birth defects. In our study, however, the IVF group appeared more susceptible to the effects of air pollution exposure compared to the ICSI group.

Overall, the effects of air pollution on birth outcomes remain inconsistent. It should be noted that the previous studies using data from spontaneous pregnancies might not be appropriate for comparison with this study. In addition, studies on the effects of air pollution exposure during the very early stages of IVF pregnancy (first two weeks) on birth outcomes are very limited.

### Potential mechanisms

Oxidative stress is one potential mechanism explaining the effects air pollution exposure on adverse birth outcomes. Oxidative stress arises from an excess of reactive oxygen species (ROS) that surpasses the protective capabilities of antioxidant mechanisms in living systems (e.g., cells, tissues, fluids) [3]. Air pollution exposure induces oxidative stress, which can harm placental function, thereby reducing the nutrients and oxygen delivered to the fetus [51]. Therefore, exposure to air pollution during prenatal periods may affect the fetal growth, potentially leading to adverse birth outcomes [43]. Furthermore, air pollutants have the potential to reach the foetus via transplacental transfer, further underscoring the complexity of these mechanisms and their impact on fetal development [23].

The germinal stage of embryonic development (14 days from conception) is highly susceptible to oxidative stress. Adverse effects on germinal development can compromise fetal development [47]. The IVF/ICSI population may be more vulnerable to oxidative stress than those conceived spontaneously. This increased vulnerability is due to the direct exposure of the developing embryo to oxidative stress sources in the embryo culture environment before transfer to the uterus. These sources include visible light, plastic IVF consumables released volatile organic compounds (VOCs), and additives in the culture media [2]. In our findings, accordingly, during window 3 (14 days embryo transfer) the very early stage of embryonic development, both adjusted and crude analysis indicates that NO_2_ were significantly increased the risk of low birthweight and SGA. PM_10_ in window 3 significantly associated with the risk of preterm birth.

### Clinical implications

The results suggest that exposure to air pollution during the very early stage of pregnancy (14 days after conception) may represent a critical window of susceptibility and is associated with an increased risk of adverse birth outcomes.

The IVF/ICSI population is inherently more susceptible to adverse birth outcomes than those conceived spontaneously [44]. However, this study found that even population living in areas with low levels of air pollution still exhibited an increased risk of adverse birth outcomes. This suggests that even low-level air pollution may further elevate the risk of adverse birth outcomes of IVF/ICSI conceptions. In addition to adverse birth outcomes, congenital malformations are also a critical concern in the IVF/ICSI births. Future research could include both naturally conceived and IVF/ICSI conceived births from the same geographic location, allowing for a direct comparison of the risk of those adverse birth outcomes attributable to environmental exposure other than air pollution (e.g., UV exposure, temperature, diet, and noise) between the two groups.

By implementing stricter air pollution control regulations, further protection can be offered to maternal and child health. Although this study does not include births from spontaneous conception, it is advisable for these women to also avoid exposure to high levels of air pollution during the gestational period, particularly in the very early stages of pregnancy.

## Conclusions

The findings indicate that exposure to air pollution during the early stages of IVF/ICSI pregnancy may contribute to an increased risk of adverse birth outcomes. Subgroup analysis revealed that ambient air pollution exposure might have a more pronounced negative impact on the IVF group compared to the ICSI group. The limited availability of relevant studies and the relatively small size of our dataset underscore the necessity for further research involving larger population samples. Expanding the dataset and conducting additional studies would enhance the understanding of how air pollution differentially affects IVF and ICSI pregnancies and help to establish more robust conclusions.

## Supplementary Information

Supplementary Material 1

## Data Availability

No datasets were generated or analysed during the current study.

## Appendix

**Appendix Table 1:** The ADMS-Urban model inputs for period 2010 to 2019. National atmospheric emission inventory (NAEI) [40] data available at: https://naei.beis.gov.uk/ (Accessed: 10 January 2024). Meteorological data is downloaded from the worldmet package in R which provides measured wind speed, wind direction, cloud cover, precipitation, relative humidity, and temperature from the local airport

Model input	Data source
Meteorological data	Hourly measured data from the local Airport
Background data	Measured air pollution data from Bush estate (NO_2_) and Auchencorth Moss (PM_2.5_ and PM_10_) rural monitoring sites
Grid emission inventory	National atmospheric emission inventory (NAEI) 1km^2^ emission inventory
Large point sources	NAEI industry point sources

**Appendix Table 2:** IVF patients’ social class classification defined by the National Records of Scotland [36]. Available at: https://www.nrscotland.gov.uk/files/statistics/vital-events/occupation-social-classification-background-jan-2015.pdf (Accessed: [23rd April 2024])

Code	Description
0	Large employers and higher managerial occupations
1	Higher professional occupations
2	Lower managerial and professional occupations
3	Intermediate occupations
4	Small employers and own account workers
5	Lower supervisory and technical occupations
6	Semi-routine occupations
7	Routine occupations
8	Never worked and long term unemployed
9	Students, not stated or not classifiable

## References

[1] Adanikin A, Lawlor DA, Pell JP, Nelson SM, Smith GC, Iliodromiti S. Association of birthweight centiles and early childhood development of singleton infants born from 37 weeks of gestation in Scotland: A population-based cohort study. PLoS Med. 2022;19(10): e1004108. 36219591 10.1371/journal.pmed.1004108 PMC9553050

[2] Agarwal A, Maldonado Rosas I, Anagnostopoulou C, Cannarella R, Boitrelle F, Munoz LV, Finelli R, Durairajanayagam D, Henkel R, Saleh R. Oxidative stress and assisted reproduction: a comprehensive review of its pathophysiological role and strategies for optimizing embryo culture environment. Antioxidants. 2022;11(3):477. 35326126 10.3390/antiox11030477 PMC8944628

[3] Aitken RJ. Impact of oxidative stress on male and female germ cells: implications for fertility. Reproduction. 2020;159(4):R189–201. 31846434 10.1530/REP-19-0452

[4] Barker DJ, Bull AR, Osmond C, Simmonds SJ. Fetal and placental size and risk of hypertension in adult life. BMJ. 1990;301(6746):259–62. 2390618 10.1136/bmj.301.6746.259 PMC1663477

[5] Barker DJP, Godfrey KM, Osmond C, Bull A. The relation of fetal length, ponderal index and head circumference to blood pressure and the risk of hypertension in adult life. Paediatr Perinat Epidemiol. 1992;6(1):35–44. 1553316 10.1111/j.1365-3016.1992.tb00741.x

[6] Balli M, Cecchele A, Pisaturo V, Makieva S, Carullo G, Somigliana E, Paffoni A, Vigano P. Opportunities and limits of conventional IVF versus ICSI: it is time to come off the fence. J Clin Med. 2022;11(19):5722. 36233589 10.3390/jcm11195722 PMC9572455

[7] Bateson TF, Wright JM. Regression calibration for classical exposure measurement error in environmental epidemiology studies using multiple local surrogate exposures. Am J Epidemiol. 2010;172(3):344–52. 20573838 10.1093/aje/kwq123

[8] Brauer M, Lencar C, Tamburic L, Koehoorn M, Demers P, Karr C. A cohort study of traffic-related air pollution impacts on birth outcomes. Environ Health Perspect. 2008;116(5):680–6. 18470315 10.1289/ehp.10952 PMC2367679

[9] Bonduelle M, Liebaers I, Deketelaere V, Derde MP, Camus M, Devroey P, Van Steirteghem A. Neonatal data on a cohort of 2889 infants born after ICSI (1991–1999) and of 2995 infants born after IVF (1983–1999). Hum Reprod. 2002;17(3):671–94. 11870121 10.1093/humrep/17.3.671

[10] Carson C, Hinton L, Kurinczuk J, Quigley M. ‘I haven’t met them; I don’t have any trust in them. It just feels like a big unknown’: a qualitative study exploring the determinants of consent to use Human Fertilisation and Embryology Authority registry data in research. BMJ open. 2019;9(5):e026469. 10.1136/bmjopen-2018-026469 PMC6549633 31152033

[11] Clemens T, Turner S, Dibben C. Maternal exposure to ambient air pollution and fetal growth in North-East Scotland: A population-based study using routine ultrasound scans. Environ Int. 2017;107:216–26. 28753483 10.1016/j.envint.2017.07.018 PMC5571229

[12] Conen D, Tedrow UB, Cook NR, Buring JE, Albert CM. Birth weight is a significant risk factor for incident atrial fibrillation. Circulation. 2010;122(8):764–70. 20697028 10.1161/CIRCULATIONAHA.110.947978 PMC2927709

[13] Curran J. Exposure to traffic-related air pollution and perinatal health. National Collaborating Centre for Environmental Health. 2014.

[14] Fauser BCJM, Devroey P, Diedrich K, Balaban B, Bonduelle M, Delemarre-Van De Waal HA, Estella C, Ezcurra D, Geraedts JPM, Howles CM, Lerner-Geva L. Health outcomes of children born after IVF/ICSI: a review of current expert opinion and literature. Reprod Biomed Online. 2014;28(2):162–82. 24365026 10.1016/j.rbmo.2013.10.013

[15] Gaskins AJ, Fong KC, Abu Awad Y, Di Q, Mínguez-Alarcón L, Chavarro JE, Ford JB, Coull BA, Schwartz J, Kloog I, Souter I. Time-varying exposure to air pollution and outcomes of in vitro fertilization among couples from a fertility clinic. Environ Health Perspect. 2019;127(7): 077002. 31268361 10.1289/EHP4601 PMC6792363

[16] Garces A, Perez W, Harrison MS, Hwang KS, Nolen TL, Goldenberg RL, Patel AB, Hibberd PL, Lokangaka A, Tshefu A, Saleem S. Association of parity with birthweight and neonatal death in five sites: The Global Network’s Maternal Newborn Health Registry study. Reprod Health. 2020;17:1–7. 33334362 10.1186/s12978-020-01025-3 PMC7745358

[17] Ghazi T, Naidoo P, Naidoo RN, Chuturgoon AA. Prenatal air pollution exposure and placental DNA methylation changes: Implications on fetal development and future disease susceptibility. Cells. 2021;10(11):3025. 34831248 10.3390/cells10113025 PMC8616150

[18] Graubard BI, Korn EL. Regression analysis with clustered data. Stat Med. 1994;13(5–7):509–22. 8023032 10.1002/sim.4780130514

[19] Herrick EJ, Bordoni B. 2023. ‘Embryology, Placenta’, StatPearls. Treasure Island (FL): StatPearls Publishing. Available at: https://www.ncbi.nlm.nih.gov/books/NBK537087/ (Accessed: 1 June 2025). 31869098

[20] Ho TH, Van Dang C, Pham TTB, Hien TT, Wangwongwatana S. Ambient particulate matter (PM2. 5) and adverse birth outcomes in Ho Chi Minh City, Vietnam. Hyg Environ Heal Adv. 2023;5:100049.

[21] Huang C, Nichols C, Liu Y, Zhang Y, Liu X, Gao S, Li Z, Ren A. Ambient air pollution and adverse birth outcomes: a natural experiment study. Popul Health Metrics. 2015;13:1–7. 10.1186/s12963-015-0050-4 PMC4506631 26190943

[22] ICMART. ICMART World Report 2019. 2019. Available at: https://www.icmartivf.org/wp-content/uploads/ICMART-worldreport_2019_preliminary.pdf. Accessed 23 Jan 2025.

[23] Kannan S, Misra DP, Dvonch JT, Krishnakumar A. Exposures to airborne particulate matter and adverse perinatal outcomes: a biologically plausible mechanistic framework for exploring potential effect modification by nutrition. Environ Health Perspect. 2006;114(11):1636–42. 17107846 10.1289/ehp.9081 PMC1665414

[24] Knop MR, Geng TT, Gorny AW, Ding R, Li C, Ley SH, Huang T. Birth weight and risk of type 2 diabetes mellitus, cardiovascular disease, and hypertension in adults: a meta-analysis of 7 646 267 participants from 135 studies. J Am Heart Assoc. 2018;7(23): e008870. 30486715 10.1161/JAHA.118.008870 PMC6405546

[25] Koukoura O, Sifakis S, Spandidos DA. DNA methylation in the human placenta and fetal growth (review). Mol Med Rep. 2012;5(4):883–9. 22294146 10.3892/mmr.2012.763 PMC3493070

[26] Krisher RL. ‘The effect of oocyte quality on development’, Journal of Animal Science. 2004;82(Suppl. E):E14–E23. 10.2527/2004.8213_supplE14x 15471793

[27] LaPointe S, Lee JC, Nagy ZP, Shapiro DB, Chang HH, Wang Y, Russell AG, Hipp HS, Gaskins AJ. ‘Air pollution exposure in vitrified oocyte donors and male recipient partners in relation to fertilization and embryo quality’, Environment International. 2024. 10.1016/j.envint.2024.109147 PMC11890188 39547088

[28] Lash TL. The harm done to reproducibility by the culture of null hypothesis significance testing. Am J Epidemiol. 2017;186(6):627–35. 28938715 10.1093/aje/kwx261

[29] Lee BE, Ha EH, Park HS, Kim YJ, Hong YC, Kim H, Lee JT. Exposure to air pollution during different gestational phases contributes to risks of low birth weight. Hum Reprod. 2003;18(3):638–43. 12615838 10.1093/humrep/deg102

[30] Liang L, Gong P. Urban and air pollution: a multi-city study of long-term effects of urban landscape patterns on air quality trends. Sci Rep. 2020;10:18618. 33122678 10.1038/s41598-020-74524-9 PMC7596069

[31] Liu HJ, Liu PC, Hua J, Zhao Y, Cao J. Placental weight and size in relation to fetal growth restriction: a case-control study. J Matern Fetal Neonatal Med. 2021;34(9):1356–60. 31234675 10.1080/14767058.2019.1636371

[32] Litzky JF, Boulet SL, Esfandiari N, Zhang Y, Kissin DM, Theiler RN, Marsit CJ. Effect of frozen/thawed embryo transfer on birthweight, macrosomia, and low birthweight rates in US singleton infants. Am J Obstet Gynecol. 2018;218(4):433-e1. 10.1016/j.ajog.2017.12.223 PMC5878119 29291410

[33] Luke B, Keith LG. The contribution of singletons, twins and triplets to low birth weight, infant mortality and handicap in the United States. J Reprod Med. 1992;37(8):661–6. 1432978

[34] Mansournia MA, Nazemipour M, Naimi AI, Collins GS, Campbell MJ. Reflection on modern methods: demystifying robust standard errors for epidemiologists. Int J Epidemiol. 2021;50(1):346–51. 33351919 10.1093/ije/dyaa260

[35] Melody S, Wills K, Knibbs LD, Ford J, Venn A, Johnston F. Adverse birth outcomes in Victoria, Australia in association with maternal exposure to low levels of ambient air pollution. Environ Res. 2020;188: 109784. 32574853 10.1016/j.envres.2020.109784

[36] National Records of Scotland (2015). Available at: https://www.nrscotland.gov.uk/files/statistics/vital-events/occupation-social-classification-background-jan-2015.pdf (Accessed: [23rd April 2024])

[37] National Records of Scotland. (2020). Postcode polygon map. Available at: https://www.nrscotland.gov.uk/statistics-and-data/geography/nrs-postcode-extract (Accessed: [01 April 2024]).

[38] National Records of Scotland. (2021) Settlements and Localities Mid-2020. Available at: https://www.nrscotland.gov.uk/statistics-and-data/statistics/statistics-by-theme/population/population-estimates/settlements-and-localities/mid-2020 (Accessed: [20th April 2024]).

[39] National Records of Scotland. (2023) Births in Scotland. https://publichealthscotland.scot/publications/births-in-scotland/births-in-scotland-year-ending-31-march-2023/ (Accessed: [5th April 2024]).

[40] National Atmospheric Emissions Inventory. (2024). Available at: https://naei.beis.gov.uk/ (Accessed: 10 January 2024).

[41] Osmond C, Barker DJ. Fetal, infant, and childhood growth are predictors of coronary heart disease, diabetes, and hypertension in adult men and women. Environ Health Perspect. 2000;108(suppl 3):545–53. 10.1289/ehp.00108s3545 PMC1637808 10852853

[42] OpenStreetMap contributors (2024). OpenStreetMap. OpenStreetMap Foundation. Available as open data under the Open Data Commons Open Database License (ODbL) at https://www.openstreetmap.org/#map=6/54.910/-3.432 (Accessed: 21 Jun 2024).

[43] Park SK, O’Neill MS, Vokonas PS, Sparrow D, Schwartz J. Effects of air pollution on heart rate variability: the VA normative aging study. Environ Health Perspect. 2005;113(3):304–9. 15743719 10.1289/ehp.7447 PMC1253756

[44] Pandey S, Shetty A, Hamilton M, Bhattacharya S, Maheshwari A. Obstetric and perinatal outcomes in singleton pregnancies resulting from IVF/ICSI: a systematic review and meta-analysis. Hum Reprod Update. 2012;18(5):485–503. 22611174 10.1093/humupd/dms018

[45] Rizk B. 2008. Infertility and assisted reproduction.

[46] Rutledge JC. Developmental toxicity induced during early stages of mammalian embryogenesis. Mutation Research/Fundamental and Molecular Mechanisms of Mutagenesis. 1997;396(1–2):113–27. 9434863 10.1016/s0027-5107(97)00178-4

[47] Swain N, Moharana AK, Jena SR, Samanta L. Impact of oxidative stress on embryogenesis and fetal development. In: Oxidative Stress and Toxicity in Reproductive Biology and Medicine: A Comprehensive Update on Male Infertility, vol. II. Cham: Springer International Publishing; 2022. p. 221–41. 10.1007/978-3-031-12966-7_13 36472825

[48] Scotland. National Infertility Group, 2016. National Infertility Group Report: March 2016. Scottish Government. https://www.gov.scot/publications/national-infertility-group-report-january-2013/ (Accessed: [23rd May 2024])

[49] Slama R, Thiebaugeorges O, Goua V, Aussel L, Sacco P, Bohet A, Forhan A, Ducot B, Annesi-Maesano I, Heinrich J, Magnin G, Schweitzer M, Kaminski M, Charles MA. Maternal personal exposure to airborne benzene and intrauterine growth. Environ Health Perspect. 2012;120(3):410–5. 10.1289/ehp.0800465 PMC2721878 19672414

[50] Spiegelman D. Regression calibration in air pollution epidemiology with exposure estimated by spatio-temporal modeling. Environmetrics. 2013;24(8):521–4. 29081677 10.1002/env.2249 PMC5659389

[51] Tapia VL, Vasquez BV, Vu B, Liu Y, Steenland K, Gonzales GF. Association between maternal exposure to particulate matter (PM2. 5) and adverse pregnancy outcomes in Lima, Peru. J Expo Sci Environ Epidemiol. 2020;30(4):689–697. 10.1038/s41370-020-0223-5 PMC7853153 32355212

[52] Textor J, Van der Zander B, Gilthorpe MS, Liśkiewicz M, Ellison GT. Robust causal inference using directed acyclic graphs: the R package ‘dagitty.’ Int J Epidemiol. 2016;45(6):1887–94. 28089956 10.1093/ije/dyw341

[53] Tennant PW, Murray EJ, Arnold KF, Berrie L, Fox MP, Gadd SC, Harrison WJ, Keeble C, Ranker LR, Textor J, Tomova GD. Use of directed acyclic graphs (DAGs) to identify confounders in applied health research: review and recommendations. Int J Epidemiol. 2021;50(2):620–32. 33330936 10.1093/ije/dyaa213 PMC8128477

[54] Thomson K, Moffat M, Arisa O, Jesurasa A, Richmond C, Odeniyi A, Bambra C, Rankin J, Brown H, Bishop J, Wing S, McNaughton A, Heslehurst N. Socioeconomic inequalities and adverse pregnancy outcomes in the UK and Republic of Ireland: a systematic review and meta-analysis. BMJ Open. 2021;11(3). 10.1136/bmjopen-2020-042753 PMC7959237 33722867

[55] Thompson E, Kassa GM, Fite RO, Pons-Duran C, Goddard FG, Worku A, Haneuse S, Hunegnaw BM, Bekele D, Alemu K, Taddesse L. Birth outcomes and survival by sex among newborns and children under 2 in the Birhan Cohort: a prospective cohort study in the Amhara Region of Ethiopia. BMJ Glob Health. 2024;9(8): e015475. 10.1136/bmjgh-2024-015475 PMC11331882 39137954

[56] van den Hooven EH, Pierik FH, de Kluizenaar Y, Willemsen SP, Hofman A, van Ratingen SW, Zandveld PY, Mackenbach JP, Steegers EA, Miedema HM, Jaddoe VW. Air pollution exposure during pregnancy, ultrasound measures of fetal growth, and adverse birth outcomes: a prospective cohort study. Environ Health Perspect. 2012;120(1):150–6. 22222601 10.1289/ehp.1003316 PMC3261932

[57] Van Cauwenberg J, De Paepe A, Poppe L. Lost without a cause: time to embrace causal thinking using Directed Acyclic Graphs (DAGs). Int J Behav Nutr Phys Act. 2023;20(1):145. 38082338 10.1186/s12966-023-01545-8 PMC10712057

[58] Wadhawan R, Oh W, Vohr BR, Wrage L, Das A, Bell EF, Laptook AR, Shankaran S, Stoll BJ, Walsh MC, Higgins RD, Eunice Kennedy Shriver National Institute of Child Health & Human Development Neonatal Research Network. Neurodevelopmental outcomes of triplets or higher-order extremely low birth weight infants. Pediatrics. 2011;127(3):e654–60. 10.1542/peds.2010-2646 PMC3304548 21357334

[59] Wardinger JE, Ambati S. ‘Placental insufficiency’, StatPearls. Treasure Island (FL): StatPearls Publishing. 2022. Available at: https://www.ncbi.nlm.nih.gov/books/NBK551634/ (Accessed: 1 June 2025). 33085318

[60] Xu X, Sharma RK, Talbott EO, Zborowski JV, Rager J, Arena VC, Volz CD. PM10 air pollution exposure during pregnancy and term low birth weight in Allegheny County, PA, 1994–2000. Int Arch Occup Environ Health. 2011;84:251–7. 20496078 10.1007/s00420-010-0545-z

[61] Xiong X, Dickey RP, Buekens P, Shaffer JG, Pridjian G. Use of intracytoplasmic sperm injection and birth outcomes in women conceiving through in vitro fertilization. Paediatr Perinat Epidemiol. 2017;31(2):108–15. 28140471 10.1111/ppe.12339

[62] Zini A, Meriano J, Kader K, Jarvi K, Laskin CA, Cadesky K. Potential adverse effect of sperm DNA damage on embryo quality after ICSI. Hum Reprod. 2005;20(12):3476–80. 16123087 10.1093/humrep/dei266

[63] Zöller B, Sundquist J, Sundquist K, Crump C. Perinatal risk factors for premature ischaemic heart disease in a Swedish national cohort. BMJ Open. 2015;5(6): e007308. 10.1136/bmjopen-2014-007308 PMC4458615 26038357

