# Supplementary material for: Assessing ambient air pollution’s effects on birth outcomes: a Scottish IVF cohort study (2010 -2018)
Source: Environ Health. 2025 Aug 4;24:54. doi: 10.1186/s12940-025-01204-4 (PMC12323187; doi:10.1186/s12940-025-01204-4)
Supplement: Supplementary file 1 — Supplementary Material 1 [file 12940_2025_1204_MOESM1_ESM.docx]

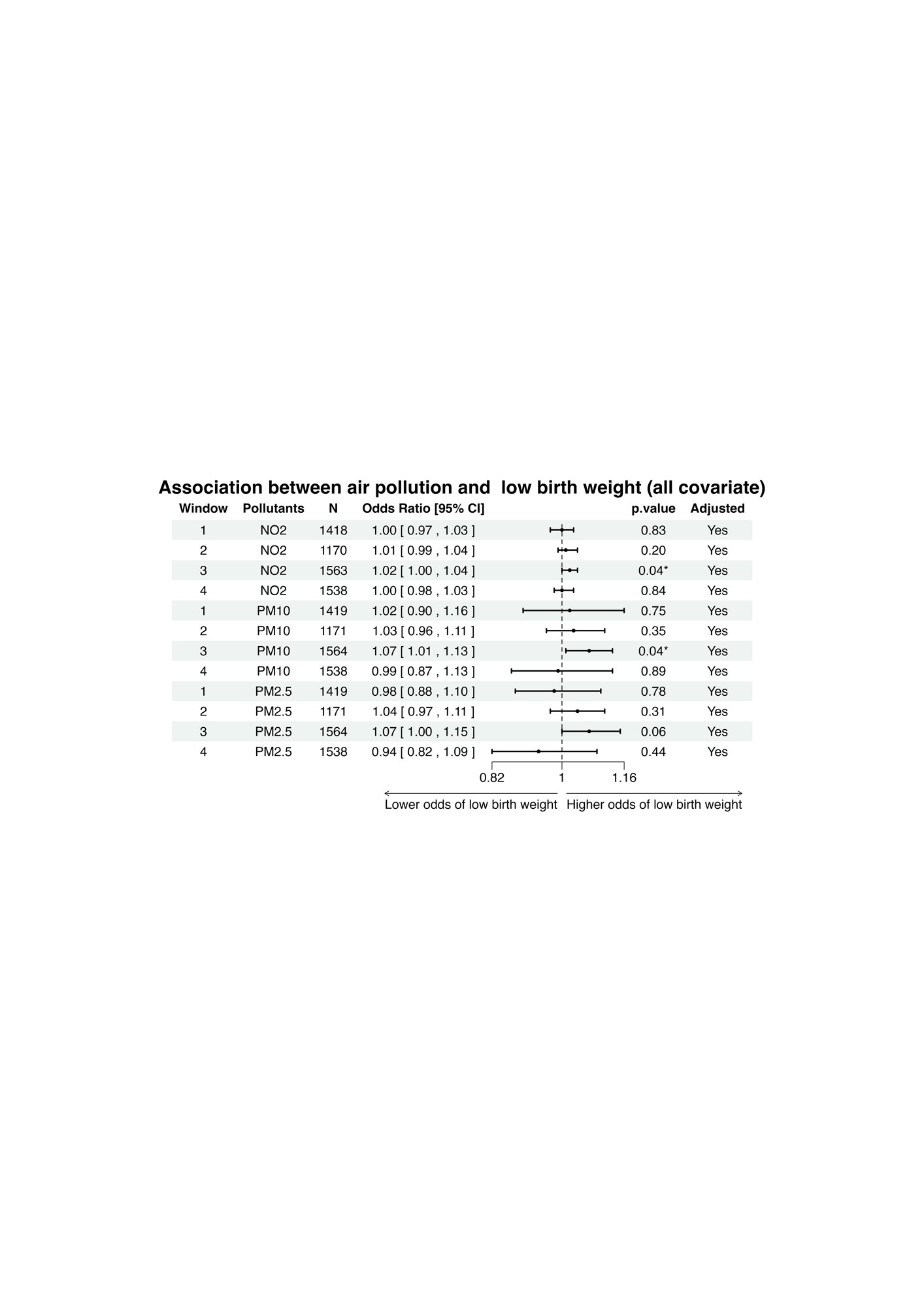


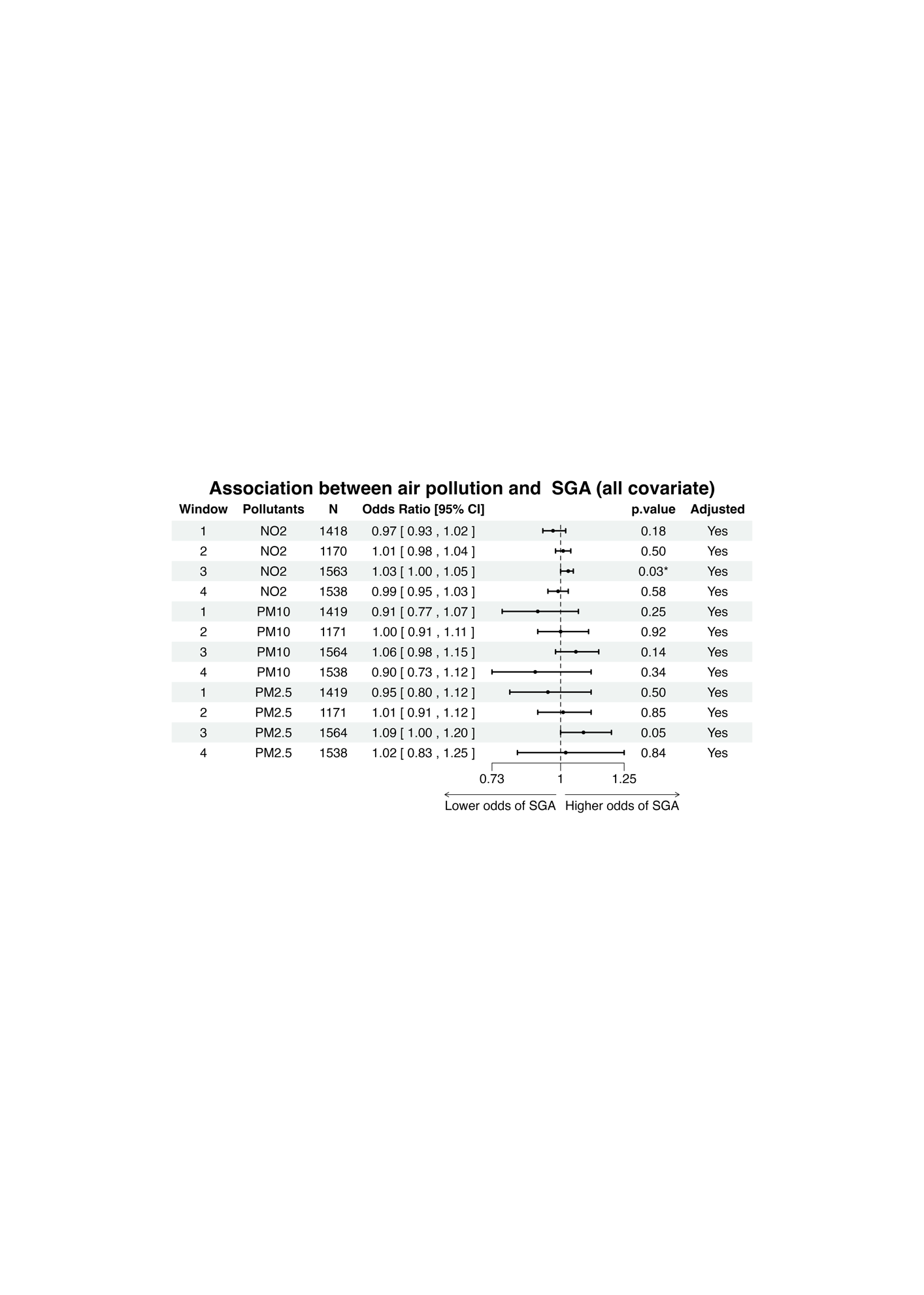


Supplementary Figure 2. Sensitivity results of alternative model setting Forest plot showing the adjusted odds ratios (ORs) with 95% confidence intervals (CIs) and p-values for the association between each 1µg⋅m^-3^ increase in air pollution concentrations and the odds of SGA across different exposure windows. "‘*’ and ‘**’ indicate p-values less than 0.05 and 0.01, respectively. N refers to the number of cases included in the analysis. The adjusted variables include the mother's occupational social class (0 - 9), infants’ sex, parity, age, cycle types (fresh or frozen), and smoking status during pregnancy (1 or 0).

Supplementary Figure 1. Sensitivity results of alternative model setting. Forest plot showing the adjusted odds ratios (ORs) with 95% confidence intervals (CIs) and p-values for the association between each 1µg⋅m^-3^ increase in air pollution concentrations and the odds of low birth weight across different exposure windows. "‘*’ and ‘**’ indicate p-values less than 0.05 and 0.01, respectively. N refers to the number of cases included in the analysis. The adjusted variables include the mother's occupational social class (0 - 9), infants’ sex, parity, age, cycle types (fresh or frozen), and smoking status during pregnancy (1 or 0).


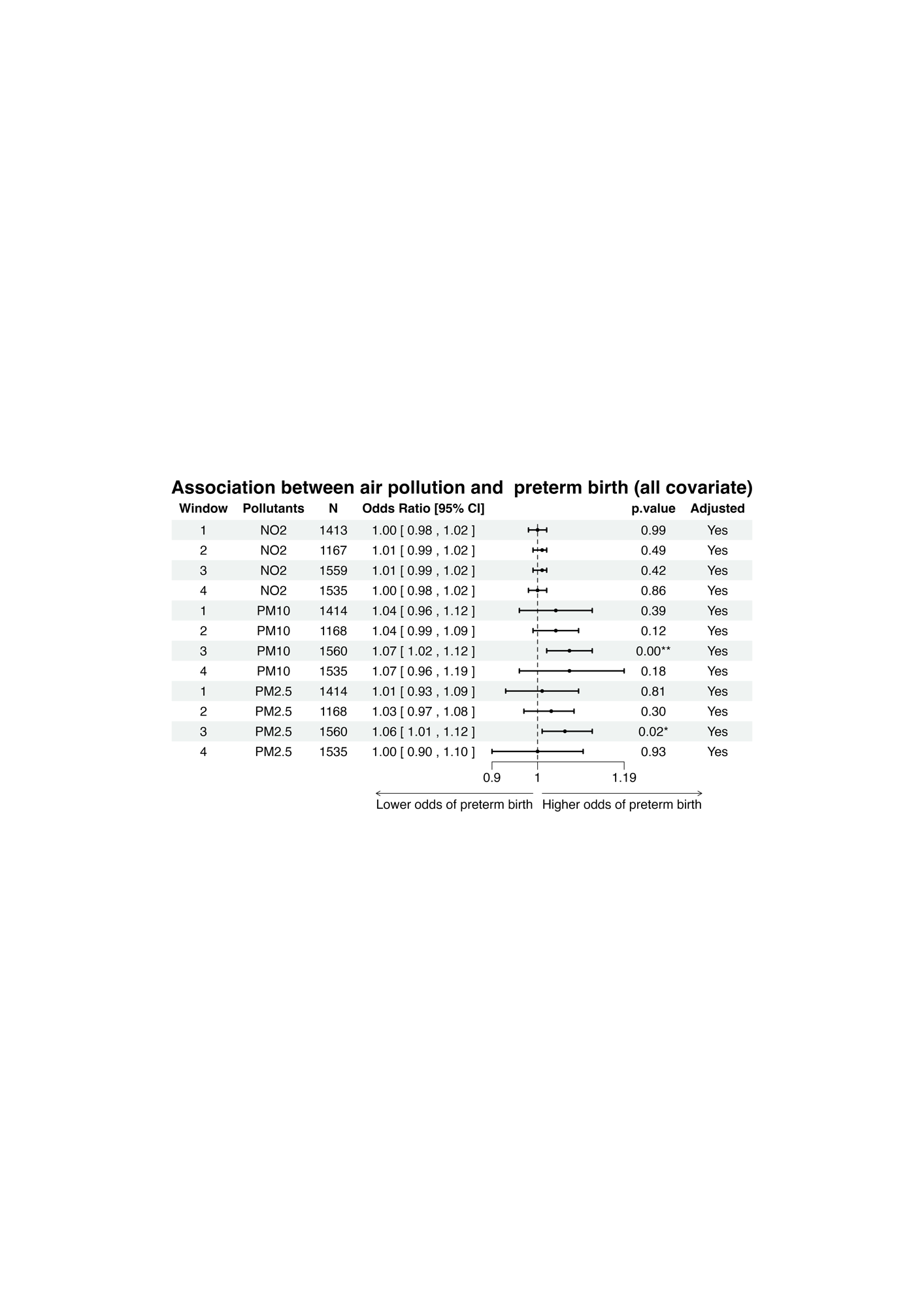


Supplementary Figure 3. Sensitivity results of alternative model setting. Forest plot showing the adjusted odds ratios (ORs) with 95% confidence intervals (CIs) and p-values for the association between each 1µg⋅m^-3^ increase in air pollution concentrations and the odds of preterm birth across different exposure windows. "‘*’ and ‘**’ indicate p-values less than 0.05 and 0.01, respectively. N refers to the number of cases included in the analysis. The adjusted variables include the mother's occupational social class (0 - 9), infants’ sex, parity, age, cycle types (fresh or frozen), and smoking status during pregnancy (1 or 0).
